# Quantitative imaging of intracellular nanoparticle exposure enables prediction of nanotherapeutic efficacy

**DOI:** 10.1038/s41467-021-22678-z

**Published:** 2021-04-22

**Authors:** Qingqing Yin, Anni Pan, Binlong Chen, Zenghui Wang, Mingmei Tang, Yue Yan, Yaoqi Wang, Heming Xia, Wei Chen, Hongliang Du, Meifang Chen, Chuanxun Fu, Yanni Wang, Xia Yuan, Zhihao Lu, Qiang Zhang, Yiguang Wang

**Affiliations:** 1 State Key Laboratory of Natural and Biomimetic Drugs, School of Pharmaceutical Sciences, Peking University, Beijing, China; 2 Beijing Key Laboratory of Molecular Pharmaceutics, School of Pharmaceutical Sciences, Peking University, Beijing, China; 3 Department of Gastrointestinal Oncology, Key Laboratory of Carcinogenesis and Translational Research (Ministry of Education), Peking University Cancer Hospital and Institute, Beijing, China

**Keywords:** Nanoparticles, Drug delivery, Imaging techniques and agents, Nanotechnology in cancer

## Abstract

Nanoparticle internalisation is crucial for the precise delivery of drug/genes to its intracellular targets. Conventional quantification strategies can provide the overall profiling of nanoparticle biodistribution, but fail to unambiguously differentiate the intracellularly bioavailable particles from those in tumour intravascular and extracellular microenvironment. Herein, we develop a binary ratiometric nanoreporter (BiRN) that can specifically convert subtle pH variations involved in the endocytic events into digitised signal output, enabling the accurately quantifying of cellular internalisation without introducing extracellular contributions. Using BiRN technology, we find only 10.7–28.2% of accumulated nanoparticles are internalised into intracellular compartments with high heterogeneity within and between different tumour types. We demonstrate the therapeutic responses of nanomedicines are successfully predicted based on intracellular nanoparticle exposure rather than the overall accumulation in tumour mass. This nonlinear optical nanotechnology offers a valuable imaging tool to evaluate the tumour targeting of new nanomedicines and stratify patients for personalised cancer therapy.

## Abstract (web summary)

Quantification of intratumoral nanoparticles internalisation in vivo is crucial but challenging. Here, the authors develop a binary ratiometric nanoreporter that can quantify internalisation and predict nanotherapeutic responses based on intracellular nanoparticle exposure.

## Introduction

Theranostic nanomedicines have made significant contributions in oncology through precise delivery of drugs into specific tissues with mitigated side-effects^1,2^. Over ten nanomedicines have been approved for cancer treatments, including PEGylated liposomal doxorubicin (Doxil/Caelyx), paclitaxel-albumin nanoparticle (Abraxane), etc^1,3^. However, cancer patients presented heterogeneous therapeutic responses to nanomedicines in clinical trials due to the extreme variability of enhanced permeability and retention (EPR) effect of solid tumours characterised by the complex tumour microenvironment^4,5^. Efforts have been made to identify cancer patients with high EPR effect (i.e. accumulation) to achieve remarkable therapeutic response^6–8^. Nevertheless, an important truth that cannot be ignored is nanomedicines with high tumour accumulation sometimes fail to achieve enhanced therapeutic response due to the inadequate target exposure (e.g. Doxil versus free doxorubicin)^9^. It is increasingly clear that drug exposure at the intracellular targets rather than the overall level in the tumour tissues acts as one of the most important factors to achieve the optimal therapeutic response^10,11^. Thus, quantifying the intracellular bioavailability of nanomedicines, including internalised amount and percentage in solid tumours would be a suitable indicator for the prediction of drug access to intracellular targets and therapeutic efficacy.

Conventional methods based on fluorescence spectroscopic^12^, mass spectrometric^13,14^ and radioactive^7,15^ detections have been extensively used to quantify the accumulation of nanomedicines in solid tumours. However, these methodologies fail to offer the information on the intracellular delivery efficiency of accumulated nanoparticles in tumour mass due to the incapability of differentiating the internalised nanoparticles from those in tumour intravascular and extracellular spaces. Several strategies such as chemical etching^16,17^, fluorescence quenching^16^ and bioluminescence reaction^18^ have been extensively leveraged to exclude the extracellularly distributed nanoparticles from endocytosis analysis, and provide two-dimensional (2D) information at cellular or ex vivo level. Despite the successful quantification of the nanoparticle internalisation in vitro, achieving real-time quantification of nanomedicines internalisation kinetics in living subjects remains a significant challenge^19^.

**Fig. 1:**
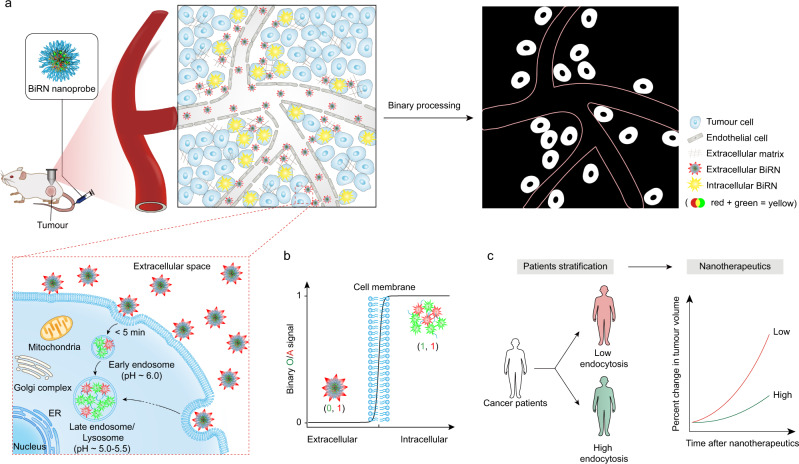
**Design and mechanism of BiRN for real-time quantitative imaging of cellular internalisation in vivo**. **a** The schematic of internalisation and activation of BiRN nanoprobes in living mice. BiRN comprises ‘OFF-ON’ and ‘always-ON’ fluorescence modules, serving as a binary pH threshold nanoreporter at pH 6.3. ‘OFF-ON’ module stays ‘OFF’ in tumour microcirculation and extracellular space (pH_e_ = 6.6–6.9), and immediately turns ‘ON’ in early endosome (pH ~6.0) after endocytosis. ‘Always-ON’ module keeps constant fluorescence intensity regardless of extra- and intracellular distribution. The ratiometric images (*F*_OFF-ON_/*F*_always-ON_, O/A) remove all the extracellular signals and only internalised nanoprobes can be quantified (white punctate) after binary processing. **b** The ratiometric O/A signal of BiRN is a binary output (0 and 1) reporting the micro-distribution of nanoparticle in the extracellular (0) and intracellular (1) space. **c** Based on BiRN, we stratify cancer patients according to endocytosis amount and predict successfully the nanotherapeutic outcomes.

Herein, we present a binary ratiometric nanoreporter (BiRN) that can unambiguously differentiate intracellularly and extracellularly located nanoparticles, permitting quantitative imaging of nanoparticle internalisation in vivo (Fig. 1a, b). We chose a well-recognised endocytosis signal, acidic early endosomal pH (pH_ee_ ~6.0) to report nanoparticle internalisation events due to the fast formation of early endosome after endocytosis (~5 min)^20–22^. Our BiRN is a two-modular nanoreporter system with a transition pH (pH_t_) at 6.3^23–26^, and each module is fluorescently encoded with a unique near-infra-red fluorophore. The ‘OFF-ON’ module keeps ‘OFF’ in tumour bloodstream and extracellular space (pH_e_ ~6.6–6.9)^27^, and immediately switches ‘ON’ upon a sudden pH drop in early endosome after endocytosis. The ‘always-ON’ module acts as an internal standard for ratiometric quantitation of nanoparticle internalisation. After binary ratiometric processing, the nanoparticles in tumour microvasculature and extracellular space were removed, allowing the real-time measurement of the cellular internalisation in living mice. Enabled by BiRN, we demonstrate the successful quantitation of nanoparticle internalisation in various animal tumour models. Furthermore, the prediction of therapeutic outcomes of cancer nanomedicines, including paclitaxel (PTX)-conjugated nanoparticle, which has identical physicochemical properties to BiRN, as well as Doxil was accomplished by patient stratification based on intracellular nanoparticle exposure in tumour mass (Fig. 1c). These capabilities make the present technology particularly potent in the comprehensive understanding of the Nano–Bio interaction in the tumour microenvironment and personalised cancer nanomedicine.

## Results

### Design and characterisation of the BiRN

**Fig. 2:**
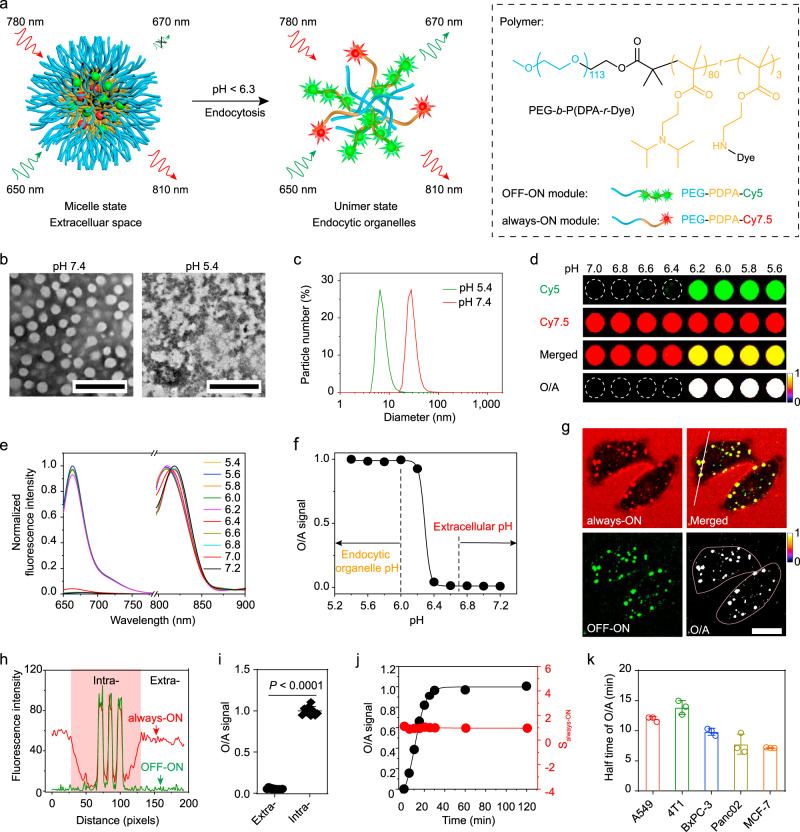
**In vitro characterisation of the BiRN**. **a** Schematic design of the BiRN. **b** Representative TEM images of micelle at pH 7.4 and 5.4. Concentration of polymer, 1 mg mL^−1^, scale bar, 200 nm. **c** Particle size distribution in pH 7.4 and 5.4 measured by dynamic light scattering. Polymer concentration, 1 mg mL^−1^. **d** Fluorescent images of the BiRN solution (100 μg mL^−1^) in different pH buffers. **e** Normalised fluorescence spectra of the BiRN in various pH solutions, which were excited at 635 and 780 nm, respectively. **f** Normalised O/A signal as a function of pH. At pH > 6.4, the value closes to 0. At pH < 6.2, the ratio turns to 1 caused by micelle dissociation. **g** Confocal images of A549 cells incubated with BiRN (100 μg mL^−1^) for 0.5 h. O/A channel images only present the internalised nanoprobes. Pink line distinguishes intracellular region from extracellular culture medium. Scale bar, 20 μm. **h** The fluorescence profiles of two modules along the white line in **g**. **i** Normalised fluorescence ratio of extra- and intracellular after cultured with BiRN for 30 min. *n* = 10 extra- or intracellular ROIs were selected for quantification. ^****^P < 0.0001, two-sided Student *t*-test. **j**, **k** The kinetics of BiRN internalisation by living cells. **j** After pulsed with BiRN at 4 °C and washed thrice, the A549 cells were chased at 37 °C for 2 h by flow cytometry. Mean ± s.d. (*n* = 3 biologically independent cell samples). The value of s.d. is too small to show. **k** The heterogeneous half-time of BiRN internalisation in various tumour cells. Mean ± s.d. (*n* *=* 3 biologically independent experiments).

We synthesised the poly(ethylene glycol)-*b*-poly(2-(diisopropyl amino)ethyl methacrylate) (PEG-*b*-PDPA) polymer with a pH_t_ at 6.3 via the atom transfer radical polymerisation method (Supplementary Fig. 1)^25^. Several dye-conjugated polymers were synthesised and divided into two groups, i.e. ‘always-ON’ modular polymer (*F*_max_/*F*_min_ < 10-fold, *F*_max_ and *F*_min_ represent the maximal and minimal fluorescence intensities at unimer and micelle states, respectively.) and ‘OFF-ON’ modular polymer (*F*_max_/*F*_min_ > 100-fold) (Supplementary Table 1 and Supplementary Figs. 2, 3). In an optimised BiRN, the dyes in the ‘OFF-ON’ and ‘always-ON’ modules should be FRET donor and acceptor, respectively (Fig. 2a). To generate the ‘always-ON’ signal, a low molar fraction of ‘always-ON’ modular polymer was used in nanoparticles to abolish homoFRET-induced fluorescence quenching (Supplementary Fig. 4). We successfully constructed four pH-sensitive BiRN nanoprobes that meet the following criteria: the fluorescence signal of ‘always-ON’ module keeps constant (*F*_max_/*F*_min_ = 0.9–1.1) at pH ranging from 5.0 to 7.4; the ‘OFF-ON’ module renders two-order magnitude signal amplification (*F*_max_/*F*_min_ > 100-fold) when pH drops below the threshold of 6.3(Supplementary Fig. 5); the pH-responsiveness of nanoparticle is very sharp (ΔpH_ON/OFF_ < 0.25) (Supplementary Table 2). In the following experiments, BiRN consisted of PDPA-Cy5 and PDPA-Cy7.5 (molar ratio, 9:1) in near-infra-red window was selected for quantitative imaging of internalisation in vivo, and BiRN comprised of PDPA-BDP and PDPA-Cy3.5 (molar ratio, 6:4) in visible window was used as an alternative for in vitro and intravital microscopic imaging studies (Supplementary Table 2).

Transmission electron microscopy (TEM) and dynamic light scattering (DLS) analyses showed that the BiRN was well-dispersed spherical structure with a diameter of 25.6 ± 2.2 nm at pH 7.4 and dissociated into unimer with a diameter of 7.6 ± 1.1 nm at pH 5.4 (Fig. 2b, c and Supplementary Fig. 6a, b). Fluorescence images and spectra of BiRN in buffers with different pH demonstrated the excellent functions of each module: ‘OFF-ON’ module had a pH transition of 6.28, a sharp pH response (ΔpH_ON/OFF_ = 0.21), and high fluorescence activation ratio of 111-fold; ‘always-ON’ module presented a constant fluorescence signal (*F*_max_/*F*_min_ = 1.03) in a broad pH range (Fig. 2d, e and Supplementary Table 2). In contrast, the fluorescence signals of two modules only exhibited negligible fluctuations towards other substances, demonstrating the high specificity of BiRN towards pH variations (Supplementary Fig. 7). The normalised ratiometric signals of the ‘OFF-ON’ to ‘always-ON’ module intensities (O/A) of BiRN close to 0 at tumour extracellular pH, whereas turn to 1 at endocytic organelle pH (Fig. 2f and Supplementary Fig. 6e), demonstrating the BiRN can be exploited to quantify cellular internalisation of nanoparticle. BiRN nanoprobes keep stable in undiluted mouse plasma over 24 h incubation as indicated by the slight change of fluorescence ratio (Supplementary Fig. 6f).

### BiRN enables the evaluation of internalisation kinetics in vitro

We directly monitor the internalisation of BiRN in A549 tumour cells without medium removal. As expected, the Cy3.5 signal homogenously distributed in the medium in ‘always-ON’ manner, whereas the BDP signal kept ‘OFF’ in the extracellular space (Supplementary Fig. 8 and Supplementary Movie 1). The BDP signal quickly turned ‘ON’ after nanoparticle internalisation as indicated by the punctate signals observed inside the cells as early as 5 min and much more green dots lighted up over time (Fig. 2g). Fluorescence of two modules showed almost perfect co-localisation inside cells, and the O/A signals presented a binary pattern as indicated by black (0) for extracellular distributed and white (1) for internalised nanoparticles (Fig. 2h, i). The binary imaging readouts were further confirmed in various tumour and non-tumour cells, including Panc02, 4T1, RAW264.7 and NIH/3T3 cells. (Supplementary Fig. 9).

Pulse-chase imaging was exploited to investigate the real-time intracellular transport of nanoparticles. Right after chasing, the nanoparticles were not internalised as shown by the strong ‘always-ON’ and undetectable ‘OFF-ON’ signals on the cell membrane (Supplementary Figs. 10 and 11). As early as 10 min incubation, the green punctate signals started to emerge in the peripherally located early endosome (pH ~6.0) derived by invagination and pinching off of plasma membrane^28^. The number of internalised fluorescent dots increased over time. After the ratiometric processing, all the cell surface-bound nanoparticles were completely wiped out and only the internalised pool can be counted. The kinetics of nanoparticle internalisation was also visualised by time-lapse fluorescence ratiometric imaging (Supplementary Movie 2). The kinetics of BiRN internalisation was further examined in various tumour cells by flow cytometry analysis (Fig. 2j). We found the half-time of nanoparticle internalisation for A549, 4T1, BxPC-3, Panc02 and MCF-7 cells is 11.89, 13.81, 9.79, 7.73 and 7.13 min, revealing a heterogeneous pattern of internalisation kinetics in different cell types (Fig. 2k and Supplementary Fig. 12). These results demonstrate the utility of the BiRN to monitor the kinetics of nanoparticle internalisation in living cells.

### BiRN quantifies nanoparticle internalisation in vivo

**Fig. 3:**
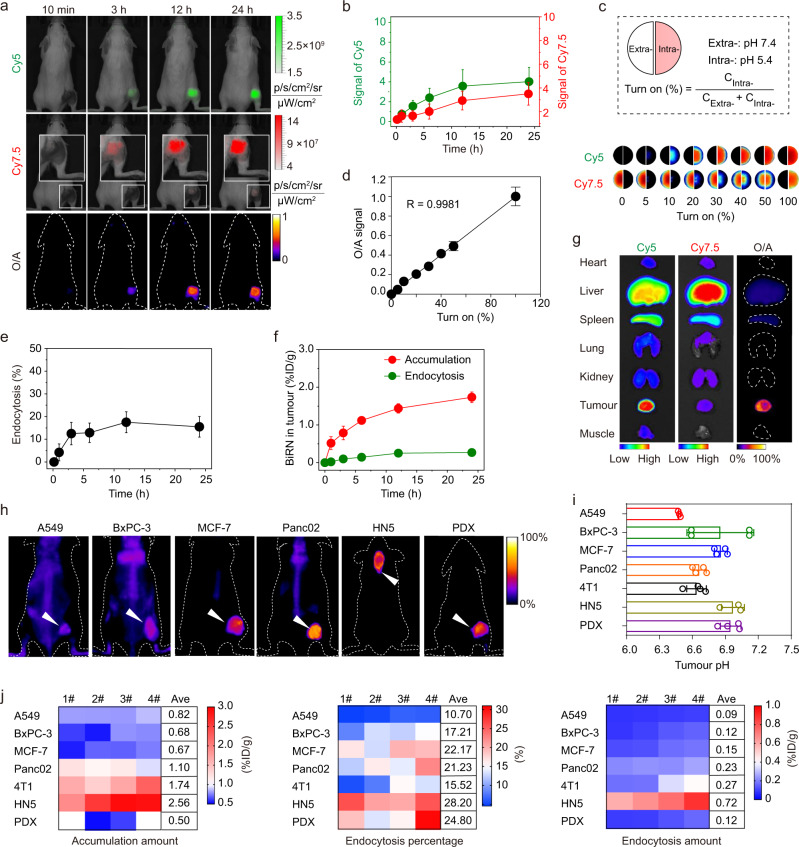
**Real-time monitoring of BiRN internalisation in vivo**. **a** 4T1 tumour-bearing mice were administrated intravenously with BiRN (20 mg kg^−1^), dual-channel fluorescence images were captured at selected time-points. Insets are magnified images. O/A channel images were generated from the ratio of Cy5 to Cy7.5 by ImageJ software. **b** Time-dependent Cy5 and Cy7.5 fluorescence intensity after BiRN injection. Mean ± s.d. (*n* = 4 biologically independent mice). **c** Establishment of the calibration curve for quantification of BiRN internalisation. Above: A diagram to illustrate the sample and calculation formula used for the preparation of calibration curve. Below: Representative fluorescence images of BiRN with different turn ON percentages. **d** O/A signal as a function of turn ON percentage. The polymer concentration is 50 μg mL^−1^. Mean ± s.d. (*n* = 3 biologically independent experiments). **e** Time-dependent endocytosis percentage of BiRN in tumour tissues after nanoparticle injection. Mean ± s.d. (*n* = 4 biologically independent mice). **f** Time-dependent intratumoural accumulation and endocytosis amount after BiRN injection. Mean ± s.d. (*n* = 4 biologically independent mice). **g** The fluorescent images of excised tumour and major organs at 24 h post-injection of BiRN. **h** The O/A images in six different tumour models (lung, pancreatic, breast, head & neck and PDX esophageal cancers). White arrows represent tumours. **i** Interstitial tumour pH (pH_e_) of different tumour types used for in vivo imaging. Mean ± s.d. (*n* = 3 biologically independent mice for A549 and HN5 models; *n* = 4 biologically independent mice for other models). **j** Heat map (*n* *=* 4 biologically independent mice) shows the heterogeneity of nanoparticle accumulation amount (left), endocytosis percentage (middle) and endocytosis amount (right) between various tumour models.

For in vivo quantification of internalisation, BiRN nanoprobes (20 mg kg^−1^) were administrated into mice bearing 4T1 breast tumours by intravenous injection. As early as 10 min post-injection, the BiRN nanoparticles rapidly accumulated into the tumour sites as indicated by the observable Cy7.5 signal, while no significant nanoparticles were endocytosed by cells within tumours due to the undetectable Cy5 and O/A signals, revealing the negligible internalisation of accumulated nanoparticle at early time-points. The Cy7.5 signals increased continuously over 24 h due to the passive accumulation of BiRN into tumour tissues through EPR effect (Fig. 3a). The Cy5 signal also increased gradually in tumour tissues with high tumour to normal tissue (T/N) contrast (Fig. 3b), indicating the efficient internalisation and activation of nanoparticle inside cells. The dual-channel fluorescence images were further collected for 7 days. The fluorescence signals of both channels gradually decreased after 48 h post-injection of BiRN, which probably due to the slow clearance of nanoparticles from mice (Supplementary Fig. 13a, b). Accordingly, O/A signal reached a maximum at 12 h post-injection followed by a slight decrease in the subsequent monitoring period (Supplementary Fig. 13c).

To quantify the nanoparticle internalisation, the calibration curve between O/A signal and internalisation percentage of the BiRN was established. A plot of O/A as a function of turn ‘ON’ (i.e. internalisation) percentage presented a linear standard curve in a concentration-independent manner (Fig. 3c, d and Supplementary Fig. 14a), which allows for quantifying internalisation efficiency regardless of nanoparticle concentrations. We further successfully established the individual standard curves (*R* > 0.9500) for main organs, such as the heart, lung, spleen, liver, kidney, tumour and muscle (Supplementary Fig. 14b), allowing the quantitative measurement of internalisation in each organs.

We then quantified the real-time nanoparticle internalisation using the established standard curves. As shown in Fig. 3e, the tumour endocytosis percentage reached a maximum at 12 h post-injection (~17.5%). The time-dependent tumour accumulation of nanoparticles was also determined by fluorometry. Based on the internalisation effficacy and accumulation results, the real-time internalised nanoparticles in tumours were successfully obtained (Fig. 3f). Results showed that about 15.5% of intratumoural nanoparticles (ca. 1.74% ID/g), that is 0.27% ID/g of nanoparticles, were internalised into the cells within tumour tissues at 24 h. The ratiometric imaging of excised organs demonstrated that nanoparticles internalisation in tumour mass is much efficient than in other organs/tissues^25^ (Fig. 3g and Supplementary Fig. 15). Despite most of the nanoprobes trapped in the mononuclear phagocyte system (i.e. liver and spleen), the internalisation efficiency of BiRN in liver and spleen was only 2.6 and 5.3%, respectively (*P* < 0.01). The tumour internalisation of BiRN was further investigated in the dose escalation study. We found the efficiency of nanoparticle internalisation into tumour tissues decreased at a high dose of 40 mg kg^−1^ probably due to the saturation of nanoparticle internalisation (Supplementary Fig. 16).

To explore the broad application in quantification of nanoparticle internalisation, we established six animal tumour models, including orthotopic HN5 head and neck cancer, multiple subcutaneous cancer (breast, pancreatic, lung) and patient-derived xenograft (PDX) oesophageal carcinoma models. In all six tumour models, we successfully quantified the BiRN internalisation in solid tumours and other organs/tissues (Fig. 3h and Supplementary Figs. 17–22). To elucidate the impact of the acidic extracellular tumour microenvironment (pH_e_) on the stability of BiRN, the pH_e_ of interstitial fluid in all tumour models was determined by needle microelectrode protocol^29^. We found that the average pH_e_ value is 6.78 ± 0.20, none of the tumour interstitial pH_e_ below the pH_t_ of BiRN that guaranteed the exclusive activation inside cells, permitting the accurate measurement of nanoparticle internalisation in solid tumours (Fig. 3i). Heat map demonstrated the heterogeneity of BiRN accumulation and endocytosis within and between tumour types (Fig. 3j). Only 10.7–28.2% of the intratumoural nanoparticles were internalised over 24 h post-injection. The tumour distribution studies showed that 0.50–2.56% ID/g of administered nanoparticles accumulated in the tumour sites. Based on distribution data, we successfully quantified that only 0.09–0.72% ID/g of nanoparticles were sequestered by intratumoural cellular compartments.

### BiRN profiles the intracellular micro-distribution of nanoparticles

**Fig. 4:**
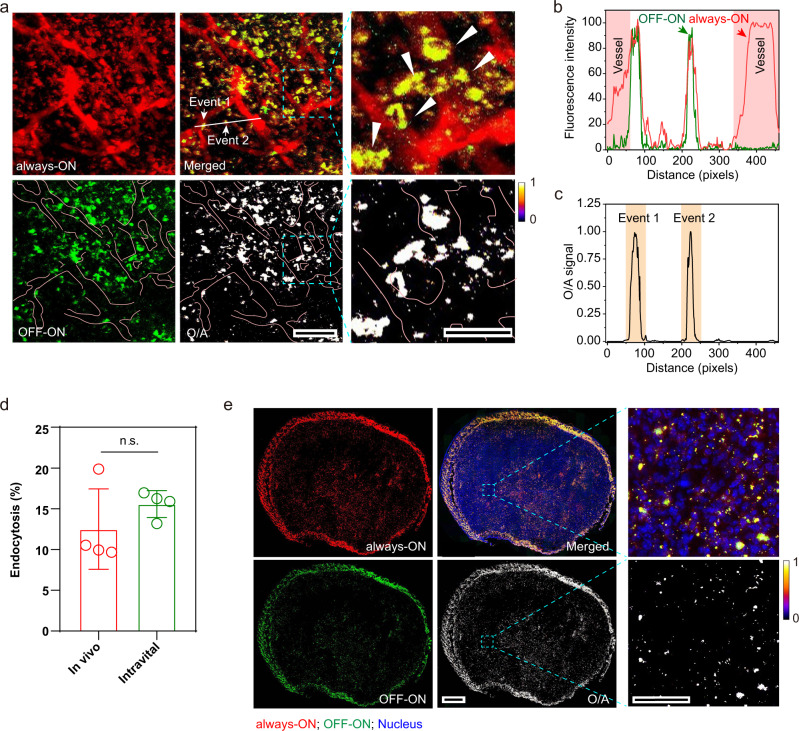
**Spatial resolution of BiRN nanoparticle microscopic distribution in vivo and ex vivo**. **a** Intravital images of tumours at 3 h post-injection of BiRN (red, Cy3.5; green, BDP; yellow, colocalization of Cy3.5 and BDP; white, internalised nanoprobes). Pink lines outline the tumour vessels, and white arrows indicate intratumoural cells taken up BiRN. Scale bar, 50 μm; insets, 25 μm. **b** The fluorescence profiles of two modules along the white line in merged channel of **a. c** O/A signal indicates endocytic events along the white line in **a**. **d** Endocytosis percentages of nanoparticles in 4T1 tumour were quantified by BiRN through two imaging methods. Data were presented as mean ± s.d. (*n* = 4 biologically independent mice), n.s. not significant, two-sided Student *t*-test. **e** Whole-mount imaging analysis of BiRN distribution and internalisation for tumour slices from 4T1 tumour-bearing mice at 24 h post-administration of BiRN. Scale bar, 800 μm. The insets are zoom-in images of squares region. Scale bar, 50 μm. (red, Cy3.5; green, BDP; blue, nucleus; yellow, colocalization of Cy3.5 and BDP; white, internalised nanoprobes).

To further investigate the microscopic internalisation of nanoparticle in living tumour-bearing mice, 4T1 tumours grown in dorsal window chambers were established to assess the function of BiRN in vivo by dual-photon intravital fluorescence microscopy. At 3 h post-injection of BiRN, the ‘always-ON’ signal distributed throughout the tumour microcirculation homogenously and extravasated in the tumour extravascular spaces, while the ‘OFF-ON’ signal was only colocalized with dot-shaped ‘always-ON’ signal in the extravascular region (Fig. 4a, b and Supplementary Movie 3). Ratiometric processing removes all the non-internalised fractions and leaves only the internalised hotspots for accurate counting (Fig. 4c). We determined that 15.6 ± 1.7% of the homing nanoparticles were internalised by 3 h (Fig. 4d), consistent with the in vivo tumour results in Fig. 3a (12.5 ± 4.9%, 3 h).

After the animal imaging, the excised tumours were collected for tissue sectioning. The fluorescence imaging of the whole-mount tumour sections (Fig. 4e) displayed an intra-individual heterogeneity in microscopic distribution and internalisation of intratumoural nanoparticles. The spatial pattern of the internalised nanoparticles in the tumours was found significantly different from that of the homing particles, exhibiting loss of the diffuse signal and leaving the highlighted punctate pattern after ratiometric processing. These data strongly support our central hypothesis that BiRN design enables the differentiation of extra- and intracellularly located nanoparticles for accurate counting of endocytic events in living animals.

### BiRN internalisation predicts therapeutic efficacy of nanomedicines

**Fig. 5:**
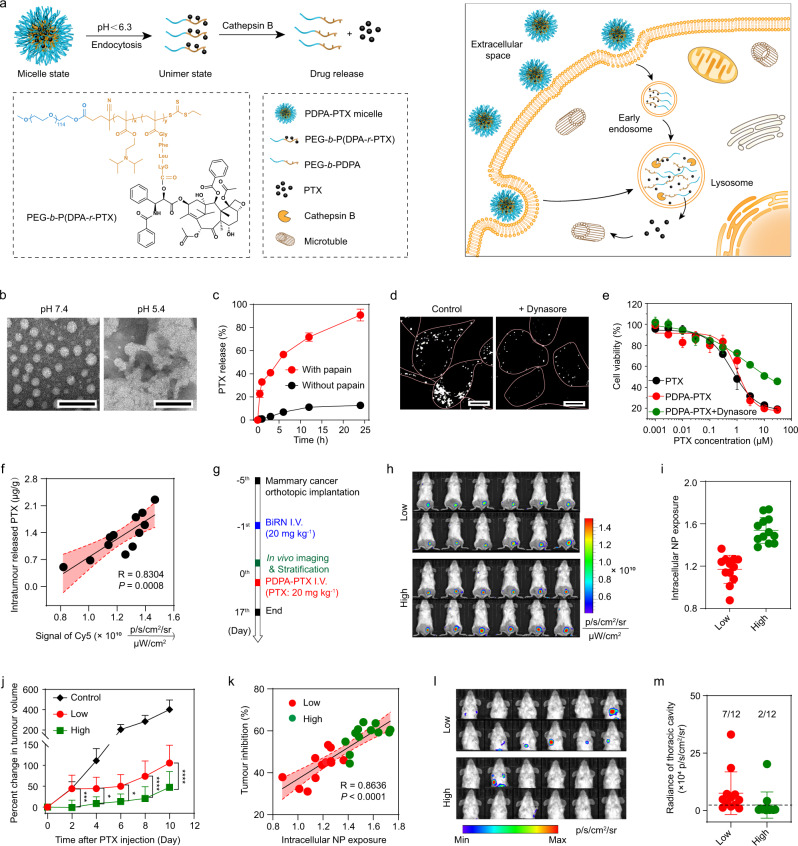
**BiRN predicts therapeutic efficacy of PDPA-PTX nanoparticle**. **a** The design of PDPA-PTX and schematic of internalisation and PTX release of PDPA-PTX in cells. **b** TEM images of PDPA-PTX at pH 7.4 and 5.4. Polymer concentration, 1 mg mL^−1^, scale bar, 100 nm. **c** In vitro PTX release profiles of PDPA-PTX in different conditions. Mean ± s.d. *(n* = 3 biologically independent experiments). **d** Confocal images of 4T1 cells treated with BiRN (100 μg mL^−1^) with or without dynasore. **e** Cytotoxicity of free PTX and PDPA-PTX micelle. Mean ± s.d. (*n* = 6 biologically independent experiments). **f** Correlation between intracellular nanoparticle exposure and intratumoural levels of released PTX at 48 h post-injection of BiRN and PDPA-PTX (PTX, 10 mg kg^−1^). *R* and *P* values were derived using a simple linear regression model. The error band in red shows the 95% confidence intervals of the fitted line by two-sided Student *t*-test analysis. **g** Experimental design for using BiRN to predict PDPA-PTX response in 4T1 orthotopic tumour model. **h** Fluorescence images of 4T1 tumour-bearing mice were captured at 24 h post-injection of BiRN (20 mg kg^−1^) (*n* = 24 biologically independent mice). **i** Intracellular nanoparticle level of 4T1 orthotopic tumour-bearing mice in **h**. Mean ± s.d. (*n* *=* 24 biologically independent mice) **j** Tumour progression in 4T1 orthotopic tumours ranked according to low and high internalisation. ^***^P *=* 0.0005 at day 2, ^*^P *=* 0.0123 at day 4, ^*^P *=* 0.0411 at day 6, ^****^P < 0.0001 at day 6 and day 8, Two-way analysis of variance (ANOVA). Mean ± s.d. (*n* = 9 mice for PBS group; *n* = 12 for other groups). **k** Correlation between intracellular nanoparticle level and 4T1 tumour progression presented as tumour inhibition percentage (*n* = 24 biologically independent mice). *R* and *P* values were derived using a simple linear regression model. The error band in red shows the 95% confidence intervals of the fitted line by two-sided Student *t*-test analysis. **l** Lung metastases imaged by in vivo BLI analysis. **m** Quantitative analysis of mice thoracic cavity signal in **l** (*n* *=* 12 biologically independent mice).

Real-time imaging of drug efficacy^30^ and toxicity^31^ has shown great potential for the evaluation of treatment response and biosafety. To investigate whether quantification of BiRN internalisation can help to predict nanotherapeutic efficacy, we introduced a pH/cathepsin B hierarchical-responsive nanomedicine recently reported by our group^32^. The nanomedicine was prepared through self-assembly of PEG-*b*-PDPA polymer-paclitaxel conjugate with enzyme cleavable Gly–Phe–Leu–Gly (GFLG) tetrapeptide linker (PEG-*b*-P(DPA-*r*-PTX), also abbreviated as PDPA-PTX. In our hypothesis, PDPA-PTX keeps micelle state in tumour microcirculation and extracellular space, but rapidly dissociated in early endosome once being internalised. After being translocated into lysosomes, the tetrapeptide linker can be cleaved by cathepsin B and the released PTX was transported into the cytoplasm, leading to effective tumour suppression (Fig. 5a).

TEM images and DLS analyses revealed that PDPA-PTX nanoparticle is a uniform spherical structure with a diameter of 41.8 ± 3.5 nm at pH 7.4, and dissociated into unimer with a diameter of 7.1 ± 0.6 nm at pH 5.4 (Fig. 5b and Supplementary Fig. 23). The enzyme-triggered release study demonstrated the half-time of PTX release was 4.52 ± 0.05 h, and >90% of PTX was released at 24 h incubation (Fig. 5c, red). In contrast, only ~10% PTX was released within 24 h in the absence of enzyme, proving the good stability of PDPA-PTX under physiological conditions (Fig. 5c, black).

To determine whether intracellular exposure of nanomedicine is the prerequisites for its anti-tumour efficacy, we performed the mechanism study of nanoparticle uptake. The results demonstrated that BiRN was endocytosed by 4T1 cells through caveolin- and dynamin-dependent endocytosis. Dynasore treatment reduced the uptake of BiRN by ~90% (Supplementary Fig. 24). The O/A images confirmed dynasore can dramatically suppress the intracellular exposure of nanoparticle (Fig. 5d). We next sought to examine the in vitro anti-tumour efficacy of PDPA-PTX. The results showed the efficacy of PDPA-PTX (IC_50_, 1.25 μM) was comparable to that of free drug (IC_50_, 0.92 μM) on a per PTX basis. In contrast, dynasore treatment led to a ~90% decrease in anti-tumour efficacy of PDPA-PTX (IC_50_, 12.87 μM) due to the marginal uptake of PDPA-PTX (Fig. 5e).

We next evaluated the potential of BiRN to predict disease progression after treatment with PDPA-PTX nanoparticle. For in vivo correlation analysis, LC-MS was performed to quantify total and released PTX contents within the tumour mass. We found that intratumoural total level of PTX is 1.80 ± 0.4% ID/g of administered PDPA-PTX, which is comparable to that of BiRN (1.74% ID/g). Furthermore, a good correlation was observed between fluorescence intensity of OFF-ON module and the released PTX content (*R* = 0.8304, *P* = 0.0008). Results demonstrated the feasibility of using intracellular nanoparticle exposure (Cy5 signal) as surrogate indicator for intracellular drug bioavailability (Fig. 5f). To test whether intracellular nanoparticle exposure could help to estimate anti-tumour efficacy of nanomedicines, mice bearing orthotopic 4T1 tumours (*n* = 24) underwent in vivo imaging at 24 h post-administration of BiRN (Fig. 5g). Fluorescence images showed endocytosis and accumulation varied vastly among individuals (Fig. 5h and Supplementary Fig. 26a). We stratified mice into low- and high-endocytosis groups according to the intracellular nanoparticles level (Fig. 5h, i). PDPA-PTX nanoparticle was administered intravenously after imaging, and tumour progression was monitored every other day (Fig. 5j and Supplementary Fig. 25). Comparing with mice in the control group with 400% change in tumour volume at day 10, the mice subjected to chemotherapy had slower tumour growth (low group, 103.9%; high group, 44.5%). Moreover, we observed significant differences (*P* = 0.026) in percentage of tumour volume change among the low- and high-endocytosis groups 2 days after treatment with PDPA-PTX, and the differences were even more significant (*P* = 0.002 at day 10) with time lapse (Fig. 5j). A good correlation (*R* = 0.8636, *P* < 0.001) between the level of intracellular nanoparticle and tumour inhibition percentage was observed on day 16 (Fig. 5k), which implies BiRN is a suitable indicator for the prediction of therapeutic efficacy of PDPA-PTX in individual subjects. In addition, the proportion of mice with lung metastases in the high group was also lower than that in low group (16.7% vs. 58.3%, Fig. 5l, m). Based on the Cy7.5 signal, we re-stratified mice bearing 4T1 tumours into low- and high-accumulation groups. Unfortunately, nanoparticle accumulation is not a good indicator for the prediction of therapeutic outcome (Supplementary Fig. 26). These results demonstrated that intracellular exposure of BiRN is a good indicator for the prediction of intracellularly bioavailable nanomedicine in tumour mass.

**Fig. 6:**
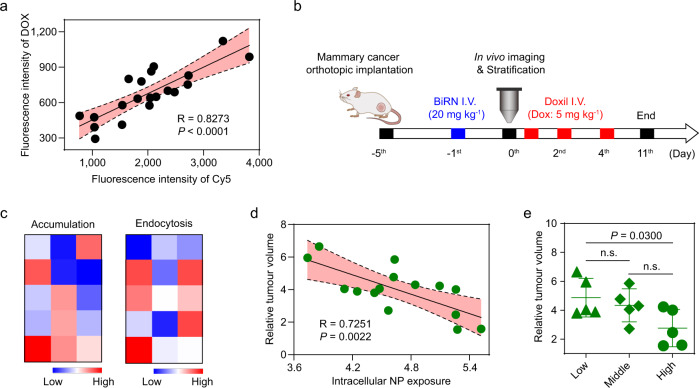
**BiRN predicts the therapeutic efficacy of Doxil**. **a** Correlation between signals of BiRN (Cy5, 20 mg kg^−1^) and Dox (5 mg kg^−1^) in tumour by flow cytometry (*n* = 20 biologically independent mice) at 24 h post-injection of BiRN and Doxil. *R* and *P* values were derived using a simple linear regression model. The error band in red shows the 95% confidence intervals of the fitted line by two-sided Student *t*-test analysis. **b** Schematic of BiRN technology to predict Doxil response in 4T1 orthotopic tumour model. **c** Heat map shows the heterogeneity of nanoparticle accumulation and endocytosis amount in 4T1 tumours for predicting Doxil response (*n* = 15 biologically independent mice). **d** Correlation between intracellular nanoparticle signal and 4T1 tumour progression presented as relative tumour volume (*n* *=* 15 biologically independent mice). *R* and *P* values were derived using a simple linear regression model. The error band in red shows the 95% confidence intervals of the fitted line by two-sided Student *t*-test analysis. **e** Intracellular nanoparticle signal stratified tumours into low, medium, or high tumour internalisation groups. Tumour response to Doxil showed significant differences between high and low group (*n* = 5 biologically independent mice). *P = 0.0300, n.s. not significant, two-sided Student *t*-test.

We proceeded to test the ability of BiRN to predict the therapeutic efficacy of other nanomedicines. PEGylated doxorubicin liposome (Doxil) was chosen as model nanomedicine for further experiments. We first performed the correlation analysis of intracellular BiRN nanoprobes and Doxil. After sequential injection of BiRN and Doxil to 4T1 tumour-bearing mice, flow cytometry analyses of cell suspensions from tumour tissues provided conclusive evidence that good correlation (*R* = 0.7996, *P* < 0.001) was observed between Cy5 and doxorubicin signals at single-cell level (Fig. 6a). Next, we evaluated the potential of BiRN to predict disease progression after treatment with Doxil (Fig. 6b). Fifty mice bearing orthotopic 4T1 tumours were imaged after intravenous administration of BiRN to measure total tumour accumulation and endocytosis (Fig. 6c). Doxil was administered intravenously after imaging, and tumours progression was monitored every other day (Supplementary Fig. 27a). We observed the relative tumour volume was linearly correlated (*R* = 0.7251, *P* = 0.0022) with endocytosis amount rather than accumulation (Fig. 6d and Supplementary Fig. 27b). We stratified mice into low, medium and high groups according to intracellular nanoparticle exposure. Similarly, we found that the high group had significantly (*P* = 0.03) better growth inhibition than low group (Fig. 6e). Therefore, BiRN reporter can be served as an effective predictor for the therapeutic outcomes of other nanomedicines.

## Discussion

We have described a two-modular nanoreporter technology for quantitative imaging of cellular internalisation in living animals. This BiRN reporter successfully converts the subtle pH change involved in the endocytic events (ΔpH < 0.6) into digitised ratiometric signal output, permitting the precision quantification of cell internalisation in vivo. While, conventional pH sensors can chemically resolve continuous variations during the endosome maturation process, but fail to accurately report the endocytic events. The present nanoreporter was successfully engineered with several critical functions that enable the directly gated counting of nanoparticle internalisation in living subjects without any sample processing. Firstly, positive cooperativity in the self-assembly of pH-responsive block copolymer drives the sharp pH response (ΔpH_ON/OFF_ < 0.25), which is essential for chemically differentiating the extracellular and endocytic pH. Secondly, the ultra-fast disassembly (<5 ms) of the nanosystem enables the real-time recording of the endocytic events without any lag time. Thirdly, our design exploits pH differences between acidic tumour microenvironment and early endosome, which is a recognised hallmark of cell internalisation regardless of tumour types. Moreover, fluorescence imaging provides a real-time, noninvasive, convenient and lower-cost paradigm for quantitative measurement of the intracellular bioavailability of nanomedicines^33^.

Our studies revealed that only 20% (median) of the nanoparticles resided in solid tumours were internalised into the cell components, while ~80% of the nanoparticles were sequestered in the acellular regions of the tumours at 24 h. More importantly, our BiRN technology demonstrated that intracellular nanoparticle exposure exhibited a strong correlation with anti-tumour efficacy of nanomedicines, whereas nanoparticle accumulation in tumour mass presented a poor correlation with therapeutic nanomedicine efficacy due to the high heterogeneity of internalisation efficiency between individual subjects. Hence, quantifying the intracellular bioavailability of nanomedicines rather than the overall accumulation in solid tumours is a suitable indicator for the prediction of anti-tumour efficacy and patient stratification for personalised treatment.

In summary, we successfully quantified the internalisation of nanoparticles that accumulated in the solid tumours at whole animal, intravital, excise tissue and tissue section levels by the BiRN technology. This internalisation nanoreporter offers a powerful tool to predict anti-tumour efficacy of different types of nanomedicines by patient stratification based on intracellular exposure of nanoparticle in tumour mass. This nanotechnology-enabled paradigm also advances a valuable tool to evaluate the tumour-targeting efficacy of new targeted nanomedicines, to uncover the dynamic Nano–Bio interaction in solid tumours.

## Methods

### Materials

The *N*-hydroxysuccinimidyl (NHS) esters of different fluorescent dyes were purchased as follows: BDP, Cy3.5, Cy5 and Cy7.5 from Lumiprobe Corporation; ICG-Sulfo-OSu from AAT Bioquest Inc; BDP650 NHS ester from Life Technologies. Ultracentrifugal filter units (MWCO = 100 kDa) were obtained from Merck Millipore. Foetal bovine serum (FBS) was purchased from Gemini Bio-products Inc. Cell culture media, penicillin-streptomycin and 0.25% trypsin-EDTA were obtained from M&C gene technology Ltd. Paclitaxel (PTX) was purchased from Dalian Meilun Biotechnology Co. Ltd. Monomers, such as 2-(diisopropylamino) ethyl methacrylate (DPA-MA) and 2-aminoethyl methacrylate (AMA) were purchased from Polysciences Company. Initiator V65 was purchased from Yuanye Biotechnology Co. Ltd. Other reagents and solvents were from Thermo Fisher Scientific or Sigma-Aldrich Inc.

### Syntheses

#### Syntheses and characterisation of dye-labelled block copolymers

PEG-*b*-PDPA-AMA_3_ copolymer was synthesised by atom transfer radical polymerisation (ATRP) method^23^. Briefly, PEG_5k_-Br (0.1 mmol), DPA-MA (8 mmol), AMA (0.3 mmol) and PMDETA (0.1 mmol) were charged into a reaction flask. Then, the above substances were dissolved by the addition of the mixed solvents of 2-propanol (2 mL) and *N*′*N*-dimethylformamide (DMF, 2 mL) into the flask. Next, CuBr (0.1 mmol) was added into the reaction flask under nitrogen atmosphere after three cycles of freeze-pump-thaw to remove oxygen, and the reaction was maintained under vacuum for 12 h at 40 °C. After the reaction, the reaction mixture was diluted with 10 mL tetrahydrofuran (THF), and the catalyst was removed by neutral aluminium oxide column. Most of the eluent was removed by rotovap. The residue was dialysed in distilled water for 24 h and then lyophilised to obtain white powder. The purified PEG-*b*-PDPA-AMA_3_ copolymer was characterised by ^1^H 400 MHz NMR and gel permeation chromatography (GPC). Molecular weight (*M*_n_ and *M*_w_) and PDI of PEG-*b*-PDPA-AMA_3_ were 22.27, 27.39 and 1.23KDa , respectively.

For fluorescent labelling of the copolymer, the dye-containing DMF solution (10 mg mL^−1^) was added into a solution of PEG-*b*-PDPA-AMA_3_ copolymer in DMF (50 mg mL^−1^). Then, the solution was stirred overnight in the dark. The purified PEG-*b*-PDPA-Dye_n_ copolymers were obtained by preparative GPC to remove the unconjugated dye molecules. The resultant dye-conjugated polymers were lyophilised and kept at −20 °C until further use. The conjugation efficiency of the dye to each polymer was determined using UV-Vis spectrophotometry. The molar feed ratios of different dyes to AMA and corresponding conjugation ratios were listed in Supplementary Table 1.

#### Syntheses of PEG-b-P(DPA-r-PTX) block copolymers

Firstly, macro chain transfer agent, PEG-CTA, was synthesised by esterification reaction. In brief, monomethoxy PEG (1.0 mM), DMAP (0.1 mM) and DCT (1.2 mM) were dissolved by the addition of anhydrous dichloromethane (DCM, 30 mL) under N_2_ atmosphere, then the solution was cooled to 0 °C. DCC (1.2 mM) in DCM (5 mL) was added into the reaction flask with continuous stirring. After stirring for 36 h at room temperature, the reaction mixture was filtrated, and then precipitated with diethyl ether twice. The product were further vacuum-dried overnight.

Next, the PEG-*b*-P(DPA-*r*-PTX) was synthesised by reversible addition-fragmentation chain transfer (RAFT) polymerisation method^32^. PEG-DCT (0.03 mmol), DPA-MA (3.7 mM), MA-GFLG-PTX (0.2 mM) and V65 (0.01 mM) were charged into a reaction flask. Then, the above substances were dissolved by the addition of anhydrous dioxane into the flask. After three cycles of freeze-pump-thaw to remove oxygen, and the polymerisation was maintained under 52 °C for 36 h. The product was purified by precipitating in large excess of cold ether, and washed three times. According to the ^1^H 400 MHz NMR, the degree of polymerisation of PDPA block was estimated to be ~76.6. The conjugation capacity of PTX was ~11.9%, which calculated by ^1^H NMR peak area relative proportion of the benzene ring in PTX and the methyl end group of the polymer.

### Preparation and characterisation of nanoparticles

#### Preparation of various monochrome nanoprobes

In a typical procedure, PEG-*b*-PDPA-Dye_n_ (*n* = 0–3) polymer (5 mg) was dissolved in methanol (1 mL). Subsequently, the solution was dropped into 10 mL distilled water under continuous sonication. The organic solvent was removed through the micro-ultrafiltration system (100 kDa) for five times. Finally, the nanoparticle solution was concentrated to 4 mg mL^−1^ (based on the polymer mass) by ultrafiltration as a stock solution for further research.

#### Characterisation of various monochrome nanoprobes

The fluorescence emission spectra of nanoprobes in PBS buffers with different pH were measured by a fluorescence spectrometer (Cary Eclipse, Agilent, USA). Taking Cy5-labelled nanoprobe for example, the nanoparticle stock solution was diluted to 0.1 mg mL^−1^ with PBS buffer of different pH. The samples were excited at 630 nm with a slit width of 5 nm, and emission spectra were collected from 650 to 720 nm. The peak intensity of emission spectra was used for the quantitative assessment of the pH-sensitive properties. The value of *R*_F_ (*F*_max_/*F*_min_), pH_t_ and ΔpH_10–90%_ are shown in Supplementary Table 1. The excitation and emission wavelength (λ_ex_/λ_em_) of dyes used to quantify pH-sensitivity are as follows: BDP (488/500–600 nm), Cy3.5 (575/590–700 nm), BDP650 (630/650–750 nm), Cy5 (630/650–750 nm), Cy7.5 (780/795–900 nm) and ICG (780/795–900 nm).

#### Preparation and characterisation of BiRN nanoprobes

After fluorophores screening of ‘OFF-ON' and ‘always-ON' modules among monochrome nanoprobes. Two dyes (BDP, Cy5) were selected as fluorophores of ‘OFF-ON' module due to their high activation ratios (*R*_F_ ≥ 100). Accordingly, four dyes (Cy3.5, BDP650, Cy7.5 and ICG) with low activation ratio (*R*_F_ ≤ 10) were chosen as potential ‘always-ON' fluorophores. Subsequently, a series of hybrid nanoparticles comprised of ‘OFF-ON' modules and potential ‘always-ON' modules with different molar ratios were prepared as mentioned above. Followed by fluorescence characterisation, the hybrid nanoparticles were diluted to 0.1 mg mL^−1^ in phosphate-buffered saline (PBS, 100 mM) with different pH from 5.4 to 7.2 (with 0.2 pH interval). The samples were successively excited with two excitation wavelengths to obtain the fluorescence emission spectra of ‘OFF-ON' and ‘always-ON' modules on a fluorescence spectrometer (Cary Eclipse, Agilent, USA).

Transmission electron microscope (JEM 1200EX, Hitachi, Japan) was used to visualise the morphology of nanoparticles at different states. The samples were diluted with Milli-Q water to 1 mg mL^−1^ or hydrochloric acid solution (pH = 5.4), and then negatively stained by 1% phosphotungstic acid. The TEM images were captured on a transmission electron microscope (JEM-1400, JEOL, Japan).

Fluorescent images of nanoprobe solution (100 μg mL^−1^) at various pH values were acquired on an IVIS in vivo imaging system (Lumina Series III, PerkinElmer, USA) using auto-exposure with excitation/emission band-pass filter (620 ± 10 nm/670 ± 20 nm). The appropriate filter pairs for different dyes were selected according to respective excitation and emission maximum.

The fluorescence stability of BiRN in the presence of ions, GSH, protease or ROS were determined. The fluorescence intensities of BiRN solution (100 µg/ml) in phosphate buffers (10 mM, pH = 7.4) were determined by an IVIS in vivo imaging system (Lumina Series III, PerkinElmer, USA) using auto-exposure with band-pass filters (λ_ex_/λ_em_: 620 ± 10 nm/670 ± 20 nm for Cy5, 780 ± 10 nm/845 ± 20 nm for Cy7.5) at 30 min after adding K^+^ (140 mM), Ca^2+^ (5 mM), Cl^−^ (114 mM), SO_4_^2−^ (20 mM) ions, GSH (10 mM), papain (5 μM), H_2_O_2_ (1 μM) and •OH (1 μM). K^+^, Ca^2+^, Cl^−^, SO_4_^2−^ ions, GSH, papain, H_2_O_2_ stock solutions were prepared by directly diluting commercially available KCl, CaCl_2_, NaCl, Na_2_SO_4_, GSH and papain respectively. •OH was produced from Fenton reaction between H_2_O_2_ and FeCl_2_.

The stability of nanoprobes was investigated by monitoring the change in fluorescence, which was measured on a fluorescence spectrophotometer (Cary Eclipse, Agilent, USA) or in vivo imaging station (Lumina Series III, PerkinElmer, USA). The fluorescence of nanoprobes was determined by diluting nanoprobes to a final concentration of 0.1 mg mL^−1^ with fresh mouse plasma or PBS buffer solutions with different pH and incubating at 37 °C in a humidified chamber for an indicated period. The change of fluorescence intensity ratio for the acceptor to donor or ‘OFF-ON' module to ‘always-ON' module over time was used for the stability evaluation.

#### Preparation and characterisation of PDPA-PTX micelle

Firstly, PDPA-PTX micelle comprised of PEG-*b*-P(DPA-*r*-PTX) was prepared as mentioned above. PTX release from PDPA-PTX micelle was performed by incubation of the micelle in Mcilvaine’s buffer (pH 6.0) at 37 °C in the presence of papain (5.0 µM) for 48 h. The stability of PDPA-PTX micelle in PBS buffer (pH 7.4) was monitored for 24 h at 37 °C. At pre-determined time-points, the released PTX amount in samples was analysed by HPLC (Agilent, USA) at UV wavelength of 230 nm. An Agilent SB-C18 (4.6 × 250, 5 μm) was maintained at 40 °C and a flow rate of 1 mL min^−1^ was used, with Milli-Q water (30%) and acetonitrile (70%) used as mobile phases^34^.

#### Preparation of pegylated liposomal doxorubicin

Liposomes were prepared by the traditional thin film-hydration method and DOX was loaded into the liposomes via the remote loading with an ammonium sulfate gradient. Briefly, HSPC, Chol and DSPE-mPEG_2000_ (molar ratio = 10:5:1) were dissolved in trichloromethane and subsequently the solvent was removed by rotovap at 37 °C to form a thin lipid film. The resulting film was hydrated with ammonium sulfate solution (123 mM), followed by sonication for 30 min. The prepared liposomes were passed through a Sephadex G-50 column equilibrated with PBS buffer (10 mM, pH = 7.2) to replace the extra-liposomal solution. Next, DOX·HCl was added into the eluted liposome suspension and incubated at 70 °C for 30 min. The separation of liposome from unloaded DOX was performed by chromatography using a Sephadex G-50 column. The concentration of DOX was determined at 495 nm utilising ultraviolet–visible (UV–Vis) spectrophotometer (UH5300, Hitachi, Japan) after lysis of liposomes with Triton X-100.

### In vitro studies

#### Cell culture

4T1 and MCF-7 breast carcinoma, A549 lung cancer cells, Panc02 and BxPC-3 pancreatic cancer cells, RAW264.7 macrophage and NIH/3T3 fibroblast were obtained from National Infrastructure of Cell Line Resource. HN5 head and neck cancer cell line was established from a head and neck cancer patient and provided by Dr. Jinming Gao Lab at University of Texas Southwestern Medical Centre. Above cell lines were maintained in complete medium (RPMI 1640 or DMEM with 10% FBS and antibiotics) in 5% CO_2_-containing humidified atmosphere at 37 °C.

#### Real-time live cell imaging

The cells were seeded in glass-bottom dishes and incubated for 24 h at 37 °C, 5% CO_2_ atmosphere to allow cell adherence and growth. For real-time imaging, cell nuclei were counterstained with Hoechst 33342 (5 μg mL^−1^) firstly and washed with PBS. Subsequently, confocal images were captured by a confocal microscope (A1R-Storm, Nikon, Japan) under 60× oil objective lens using 405 nm (nuclei, Hoechst 33342), 488 nm (OFF-ON module, BDP) and 561 nm (always-ON module, Cy3.5) lasers. The DAPI (450/35), FITC (515/30 nm), TRITC (605/75 nm) emission filters were used for Hoechst 33342, BDP and Cy3.5 imaging, respectively. Image acquisition was performed as soon as the addition of the culture medium containing BiRN (100 μg mL^−1^), followed by real-time imaging for 30 min with a time interval of 1 min between frames. The raw images were processed by NIS-Elements viewer software (Nikon) and the ratiometric images were generated by ImageJ software (NIH).

#### Kinetics study of BiRN internalisation in vitro

To investigate the kinetics of BiRN internalisation by living cells, time-resolved qualitative and quantitative experiments were separately measured in a Nikon confocal microscope (A1R-Storm, Nikon, Japan) and a flow cytometry (CytoFLEX LX, Beckman, USA).

For confocal imaging, after counterstained with Hoechst 33342 (5 μg mL^−1^), the cells were precooled on ice for 10 min, pulsed with BiRN solution (100 μg mL^−1^) at 4 °C for 10 min, washed thrice with ice-cold PBS, and chased by imaging at 37 °C for 30 min with a time interval of 3 min. Three channels, time-lapse images were collected using Nikon confocal microscope equipped with a 60× objective lens. Acquisition settings were optimised for each channel to prevent photobleaching of fluorophores. Frames were extracted using the NIS-Elements viewer software (Nikon) and the ratiometric images were generated by ImageJ software (NIH).

For quantitative analysis, the cell suspension obtained by trypsinization were put on ice for 10 min, followed by centrifugation and re-suspension in BiRN solution (100 μg mL^−1^) at 4 °C. After pulsed for 10 min (cell binding) and washed three times, the samples were chased at 37 °C (internalisation) for 2 h by measuring the fluorescent intensity of each channel for single cell on flow cytometer (Beckman Coulter) with 488 and 561 nm laser. The 488 and 561 nm lasers were used to excite BDP and Cy3.5 respectively, and appropriate compensation settings prevented crosstalk between channels. CytExpert 2.3 software was used for quantitative data collection.

#### Study on the uptake mechanism of BiRN

4T1 cells (5 × 10^5^ cells/well) were seeded into 6-well plates and cultured until adherence. To explore the cellular uptake mechanism, the cells were pre-treated with various endocytosis inhibitors, including 25 mM EIPA, 5 mM chlorpromazine, 15 mM nystatin or 40 mM dynasore for 0.5 h. Then, 100 μg mL^−1^ BiRN was added into each well and incubated for 2 h. The cells were collected by trypsinization, washed thrice and analysed on a flow cytometry.

#### Cytotoxicity evaluation

The cytotoxicity of free PTX and PDPA-PTX micelle against 4T1 cells in the absence and presence of dynasore was measured by MTT assay. 4T1 cells (5 × 10^3^ cells/well) were seeded into 96-well plates and incubated overnight. Then, the cells were pre-incubated with 40 mM dynasore for 0.5 h. Free PTX or PDPA-PTX micelle were added at final PTX concentrations of 0.001–30 μM in media and incubated for 1 h at 37 °C, followed by the replacement of fresh medium. Finally, cell viability was quantitatively evaluated by MTT assays after another 70 h incubation.

### In vivo studies

#### Animal models

All animal care and procedures were carried out in compliance with ethical compliance and approval by the Animal Ethics Committee of Peking University. Female *nu/nu* nude, BALB/c and NOD SCID mice (18–22 g) were purchased from Vital River Laboratory Animal Centre (China). Animals were housed in groups of 4–5 per cage, maintained at a temperature of ~25 °C in a humidity-controlled environment with a 12 h light/dark cycle. The BALB/c mice were subcutaneously inoculated with 4T1 tumour cells (1 × 10^6^ cells per mouse) on their right flanks. Two to three weeks after implantation (once tumour reached about 200 mm^3^ in volume), the tumour-bearing mice were subjected to imaging studies. To demonstrate the broad application of BiRNs for real-time monitoring of tumour accumulation and cellular endocytosis, subcutaneous tumour models, including MCF-7 breast carcinoma, Panc02 and BxPC-3 pancreatic cancers, A549 lung carcinoma, were developed. Orthotopic tumour models, including HN5 head-neck cancer, and 4T1 mammary carcinoma were established. PDX oesophageal tumour model in NOD SICD mice was also developed. Human oesophageal tumours were obtained from Peking University Cancer Hospital. The study was approved by the ethics committee of Peking University Cancer Hospital and with the patients’ informed consent. The PDX tumours were cut into ~2 × 2 × 2 mm small fragments and surgically engrafted into right flanks of NOD SCID mice. One month after implantation (tumour volume reached ~100–150 mm^3^), the tumour-bearing mice were used for imaging studies. For the evaluation of nanotherapeutic efficacy, 1 week later (the tumours reached a volume of 50 mm^3^), 4T1 tumour-bearing mice were treated with Doxil after ratiometric imaging.

#### Establishment of the calibration curve for quantification of BiRN internalisation

For the establishment of the calibration curve, BiRN diluted in the medium with pH 7.4 and pH 5.4 were used to simulate nanoprobes with micelle state in extracellular matrix and with unimer state in endocytic organelles, respectively. Taking PBS buffer as medium, the nanoprobes with constant polymer weight were diluted separately in two kinds of pH buffers (50 μL each) to pre-calculated concentrations, and the samples with varying ‘turn on' (endocytosis) percentages (proportion of nanoparticles in pH 5.4) were obtained. The samples with 0, 5, 10, 20, 30, 40, 50 and 100% endocytosis percentages were prepared, and then placed in a black 384-well plate, followed by fluorescence imaging using an IVIS in vivo imaging system (Lumina Series III, PerkinElmer, USA). Fluorescence images were captured using auto-exposure with band-pass filters (λ_ex_/λ_em_: 620 ± 10 nm/670 ± 20 nm for Cy5 and 780 ± 10 nm/845 ± 20 nm for Cy7.5). The mean fluorescence intensities of two channel images with varying endocytosis percentages were measured using Living Image software (PerkinElmer). The ratio value as a function of endocytosis percentage is plotted according to the formula:

$${\text{Ratio}}=\frac{F{\text{Cy}}5,{\text{pH}}7.4+F{\text{Cy}}5,{\text{pH}}5.4}{F{\text{Cy}}7.5,{\text{pH}}7.4+F{\text{Cy}}7.5,{\text{pH}}5.4}$$

To establish a calibration curve under mimetic physiological condition, a series of tissues homogenates with two pH were prepared to dilute BiRN. Fresh tissues including the heart, lung, spleen, liver, kidney, tumour and muscle were collected immediately after tumour-bearing mice were transcardially perfused with 20 mL PBS buffer. The excised tissues were cut into small pieces, weeded out connective tissues, weighed and homogenised in PBS with pH 7.4 or pH 5.4 using a homogeniser (T10 basic Ultra-Turrax, IKA, Germany). The samples of tissue homogenates with varying endocytosis percentages were prepared following the aforementioned dilution method and imaged on an in vivo imaging system. The calibration curve of each tissue homogenate was used to calculated corresponding endocytosis percentages. BiRN was diluted in PBS and tumour homogenates with varying overall concentrations (20, 50, 100 μg mL^−1^) to evaluate the effect of nanoprobes concentrations on the slopes of calibration curves.

#### Quantification of nanoparticle accumulation in tumour by fluorescence spectrophotometry

To establish a calibration curve for quantification, fresh tumours were collected from A549 tumour-bearing mice. The excised tumours were cut into small fragments, weeded out connective tissues, weighed and homogenised in acid methanol (0.5 mL/100 mg tumour) using a homogeniser (T10 basic Ultra-Turrax, IKA, Germany). A series of standard samples with different nanoparticle concentrations (0, 0.01, 0.05, 0.1, 0.5, 1, 5, 10 μg mL^−1^) were obtained by diluting nanoparticles with tumour homogenates. The supernatant liquid obtained by centrifugation (9.6 × 10^3^x*g*) were subjected to fluorescence intensity measurement on a fluorescence spectrometer (F-7000, Hitachi, Japan). Then, a linear relationship between fluorescence intensity and concentrations were established using the linear regression method. For intratumoural nanoparticle accumulation quantification, tumours (*n* *=* 4) were dissected at pre-determined time-points post-injection of nanoparticle. The homogenates and centrifugal supernatant of tumours were prepared by the above-mentioned methods. The amount of intratumoural nanoparticles were calculated from the linear standard curves.

#### In vivo and ex vivo ratiometric fluorescence imaging

The 4T1 tumour-bearing mice (*n* = 4) received BiRN nanoprobes intravenously for ratiometric fluorescence imaging to monitor tumour accumulation and internalisation of nanoparticles. The images were captured on an IVIS in vivo imaging system (Lumina Series III, PerkinElmer, USA) with time lapse using auto-exposure with a band-pass filter (λ_ex_/λ_em_: 620 ± 10 nm/670 ± 20 nm for Cy5, 780 ± 10 nm/845 ± 20 nm for Cy7.5). To demonstrate the broad application of BiRN and compare the difference between various tumours in accumulation and endocytosis, BiRN was administrated intravenously into other six tumour models mentioned above. Then, the mice were monitored by sequential acquisition at 670 and 845 nm at designated time-points. Mice were euthanized after the last imaging session, followed by resection of the tumour and organs. The dissected tumours and organs were fluorescently imaged immediately. The images of Cy5 and Cy7.5 channels were extracted using Living Image software (PerkinElmer) and the ratiometric images were generated by ImageJ software (NIH).

#### Intravital imaging of BiRN nanoprobes distribution in vivo

For visualisation of nanoprobe distribution in living animal, female nude mice (20–22 g) were implanted with a dorsal skin window chamber following the protocol previously published^35^. 4T1 tumour cells (1 × 10^6^) were harvested in up to 20 μL of phenol red–free culture medium and injected between the dermis and the exposed fascia. Over weeks, tumours reached several millimetres in size and became vascularised. Intravital imaging was performed on a dual-photon fluorescence microscope (TCS-SP8 DIVE, Leica, Germany). Mice were anaesthetised with avertin (1.2%, w/v) on a heated microscope stage and 100 μL of the nanoprobes (40 mg kg^−1^) were administered intravenously, followed by imaging at pre-determined time points. A 25× water immersion objective (NA 1.0) was used for data collection. The nanoprobe was excited at 1000 nm and the fluorescent signals of BDP and Cy3.5 were detected at 510 and 610 nm, respectively. Image analysis was performed in Leica LAS X software (Germany).

#### Visualisation of nanoprobes in excised tumour tissues

For the micro-distribution studies of nanoprobes in tissues, BiRN at a dose of 40 mg kg^−1^ was injected intravenously into 4T1 tumour-bearing mice. At 24 h post-injection, the mice were euthanized and the tumour tissues were immediately excised, embedded in O.C.T. tissue freezing medium (Leica), and snap frozen. Cryostat sections of 10 μm thickness were thawed briefly, counterstained with Hoechst 33342 (5 μg mL^−1^) and mounted. Whole slides were scanned on an automated quantitative pathology imaging system (Vectra Polaris, PerkinElmer, USA) using DAPI, FITC and Texas red excitation filters at 20× magnification.

#### Measurement of tumour interstitial pH in vivo

pH measurements were carried out on a pH metre (FE28 Five Easy, Mettler Toledo, Switzerland) with needle electrode for pH and reference electrode (MI-407B and MI-402, Microelectrodes, USA) when tumour volume reached to 200 mm^2^. Animals were placed on a heated blanket under anaesthesia by avertin (1.2%, w/v) during the experiment. After calibration of pH metre by two pH buffers, the reference electrode (2 mm OD) was placed under the skin adjacent to tumour by a shallow incision, firstly. The needle electrode (0.75 mm OD) for pH was inserted into the centre of tumour, and was held still for a moment until pH readings stabilised. Electrodes were calibrated again at the end of each measurement. Measurements with two directions were taken at each position and three positions were selected at each tumour.

#### Prediction of 4T1 tumour response to PDPA-PTX by BiRN

Orthotopic 4T1-Luc tumour-bearing mice (*n* *=* 32) with an average tumour volume of 50 mm^3^ were fluorescently imaged at 24 h post-administration of BiRN (20 mg kg^−1^). The heterogeneity of EPR effect and internalisation amount of nanoparticles among tumours were quantified by the Cy7.5 and Cy5 signals over the entire tumour area, respectively. After imaging, three-quarters of the mice received the PDPA-PTX micelle with a single dose of 20 mg kg^−1^ PTX by intravenous administration. For tumour progression monitoring, caliper measurements were performed every two days to monitor the tumour volume (V = Length × width^2^/2). Pulmonary metastasis was detected on 17th day by bioluminescence imaging with an IVIS in vivo imaging system (Lumina Series III, PerkinElmer, USA). At the end of the experiment, the GraphPad Prism 8 was used for correlation analysis between tumour progression and tumour accumulation as well as the internalisation of nanoparticles.

#### Determination of the released and total paclitaxel content in tumour tissues

4T1 tumours (*n* = 13) with average volume of 200 mm^3^ were collected at 48 h after co-injection of BiRN (20 mg kg^−1^) and PDPA-PTX (PTX, 10 mg kg^−1^). The excised tumours were cut into small fragments, weeded out connective tissues, weighed and homogenised in pH 6.0 Mcilvaine buffer (0.3 mL/100 mg tumour) using homogeniser (T10 basic Ultra-Turrax, IKA, Germany). The resulted tumour homogenates were lysed by three times freeze-thaw. The lysate (20 µL) was divided into two parts for intratumoural total and released PTX content determination, respectively. The PTX in tumour homogenates was extracted with methanol (1:5, v/v). Docetaxel (DTX) was spiked as the internal standard before the addition of the extraction solvent. For PTX contents determination, LC-MS analysis was carried out on a Triple TOF 4600 mass spectrometer (AB SCIEX, CA) equipped with a TurboV^®^ ionisation source and LC-20AD liquid chromatography system (Shimadzu, Japan). A C18 column (2.1 × 100 mm, 3.5 μm, X Bridge Waters, USA) was maintained with a flow rate of 1 mL min^−1^ at 40 °C. Milli-Q water with 0.1% formic acid and acetonitrile with 0.1% formic acid were used as mobile phases A and B, respectively. Masses detected in ESI^+^ mode were 876.5/308.1 for free PTX and 830.6/549.4 for DTX.

#### Prediction of 4T1 tumour response to doxorubicin liposome by BiRN

Orthotopic 4T1 tumour-bearing mice (*n* = 35) with average tumour volume of 50 mm^3^ were fluorescently imaged at 24 h post-injection of BiRN (20 mg kg^−1^). The heterogeneity of EPR effect and internalisation of nanoparticle among tumours were quantified by the Cy7.5 intensity and O/A value over the entire tumour area, respectively. After imaging, the mice received the doxorubicin liposome (5 mg kg^−1^) every other day for three times by intravenously administration. For the correlation study, 20 of mice were euthanized and tumours were immediately excised. Then, tumours were cut into small fragments and subsequently incubated in 2 mL of PBS containing 0.5 mg mL^−1^ collagenase IV and 0.2 mg mL^−1^ DNase I for 30 min at 37 °C while shaking. Tumour lysate was filtered using a 40 μm cell strainer (BD Falcon) to remove large aggregates. The filtrate was washed three times with PBS, followed by lysis of red blood cells by lysis buffer (BioLegend). Finally, the tumour cell samples were subjected to flow cytometry (FACSCalibur, BD, USA) to quantify fluorescent signals of Cy5 and doxorubicin. The rest of mice were used for tumour progression monitoring. Caliper measurements were performed every two days to monitor the tumour progression by the volume (*V* = Length × width^2^/2). At the end of the experiment, the Graphpad Prism 8 software was used for correlation analysis between tumour progression and tumour accumulation as well as internalisation of nanoparticles.

### Statistics and reproducibility

TEM experiments of BiRNs and PDPA-PTX nanoparticles in different pH solution were repeated thrice independently with similar results, and one representative image from each group was shown. Confocal imaging of different cells incubated with BiRN in the absence or presence of Dynasore and pulse-chase imaging of BiRN in different cells were repeated at least three times with similar results, and a series of representative images from each group were shown. Intravital imaging of tumours post-injection of BiRN was repeated three times using biologically independent mice with similar results, and a series of representative images were shown. For the whole-mount imaging analysis of BiRN distribution and internalisation for tumour slices, the experiment was repeated thrice with similar results, a series of representative images were shown.

### Statistics and analysis

Data were expressed as mean ± s.d. Statistical analyses were performed using GraphPad Prism and Origin. Significant differences of quantitative data were analysed by conducting an independent-samples *t*-test or analysis of variance (ANOVA) depending on the number of treatment groups and distribution.

### Reporting summary

Further information on research design is available in the Nature Research Reporting Summary linked to this article.

## Supplementary information

Supplementary Information

Description of Additional Supplementary Files

Supplementary Movie 1

Supplementary Movie 2

Supplementary Movie 3

Reporting Summary

## Data Availability

The source data underlying Fig. 4d and all the mouse intervention studies are provided as a Source Data file. All the other data supporting the findings of this study are available within the Article and its Supplementary Information files and from the corresponding author upon reasonable request. A Reporting Summary for this article is available as a Supplementary Information file. Source data are provided with this paper.

## Source data

Source Data
