# Supplementary material for: Quantitative imaging of intracellular nanoparticle exposure enables prediction of nanotherapeutic efficacy
Source: Nat Commun. 2021 Apr 22;12:2385. doi: 10.1038/s41467-021-22678-z (PMC8062465; doi:10.1038/s41467-021-22678-z)
Supplement: Supplementary file 1 — Supplementary Information [file 41467_2021_22678_MOESM1_ESM.pdf]

Supplementary Information

Quantitative imaging of intracellular nanoparticle exposure enables prediction of nanotherapeutic efficacy

*Qingqing Yin[1,2], Anni Pan[2], Binlong Chen[2], Zenghui Wang[2], Mingmei Tang[2], Yue Yan[2], Yaoqi Wang[2], Heming Xia[2], Wei Chen[1], Hongliang Du[2], Meifang Chen[2], Chuanxun Fu[2], Yanni Wang[3], Xia Yuan[2], Zhihao Lu[3], Qiang Zhang[1,2], Yiguang Wang[1,2,]**

[1] State Key Laboratory of Natural and Biomimetic Drugs, School of Pharmaceutical Sciences, Peking University, Beijing 100191, China, [2] Beijing Key Laboratory of Molecular Pharmaceutics, School of Pharmaceutical Sciences, Peking University, Beijing 100191, China. [3]Department of Gastrointestinal Oncology, Key Laboratory of Carcinogenesis and Translational Research (Ministry of Education), Peking University Cancer Hospital and Institute, Beijing, China

* Corresponding authors.
E-mail address: yiguang.wang@pku.edu.cn

Table of Contents

1. Supplementary Figures....................5

Supplementary Fig. 1. Syntheses of PEG_{114}-*b*-$PDPA_{80}$-AMA_3 and dye-conjugated PEG_{114}-*b*-$PDPA_{80}$ copolymers....................5

Supplementary Fig. 2. pH-dependent fluorescence emission spectra of dye-conjugated PEG_{114}-*b*-$PDPA_{80}$ copolymers....................6

Supplementary Fig. 3. Fluorescence ratio as a function of pH for dye-conjugated PEG_{114}-*b*-$PDPA_{80}$ copolymers....................7

Supplementary Fig. 4. Characterization of always-ON modules for rational design of binary ratiometric nanoreporters....................8

Supplementary Fig. 5. The "turn-on" mechanism of BiRN probe....................9

Supplementary Fig. 6. Characterization of the binary ratiometric nanoreporter in visible window....................10

Supplementary Fig. 7. The fluorescence stability of BiRN in the presence of ions, GSH, protease or ROS....................11

Supplementary Fig. 8. Confocal images of A549 cells treated with BiRN at the indicated incubation time....................12

Supplementary Fig. 9. Confocal images of representative cells treated with BiRN for 0.5 h....................13

Supplementary Fig. 10. Kinetics study of BiRN internalization in A549 lung cancer cells. 14

Supplementary Fig. 11. Kinetics study of BiRN internalization in Panc02 pancreatic cancer cells....................15

Supplementary Fig. 12. Kinetics study of BiRN internalization in various cells....................16

Supplementary Fig. 13. Long-term monitoring of BiRN internalization in vivo....................17

Supplementary Fig. 14. Establishment of quantitative analysis method for BiRN internalization....................18

Supplementary Fig. 15. Quantification of the nanoparticle accumulation and endocytosis percentage in different organs....................19

Supplementary Fig. 16. Study on the concentration-dependence of accumulation and endocytosis in vivo....20

Supplementary Fig. 17. Real-time monitoring of tumour accumulation and cellular endocytosis of BiRN in MCF-7 breast tumour xenografts....21

Supplementary Fig. 18. Real-time monitoring of tumour accumulation and cellular endocytosis of BiRN in Panc02 pancreatic tumour xenografts. 22

Supplementary Fig. 19. Real-time monitoring of tumour accumulation and cellular endocytosis of BiRN in HN5 head & neck tumour xenografts....23

Supplementary Fig. 20. Real-time monitoring of tumour accumulation and cellular endocytosis of BiRN in A549 lung cancer xenografts....24

Supplementary Fig. 21. Real-time monitoring of tumour accumulation and cellular endocytosis of BiRN in BxPC-3 pancreatic tumour xenografts. 25

Supplementary Fig. 22. Real-time monitoring of tumour accumulation and cellular endocytosis of BiRN in PDX esophageal carcinoma model....26

Supplementary Fig. 23. Characterization of PDPA-PTX micelles....27

Supplementary Fig. 24. Endocytosis mechanism of PDPA-PTX micelles....28

Supplementary Fig. 25. Individual tumour growth curves and weight change of 4T1 tumour-bearing mice in different groups according to Cy5 signal. 29

Supplementary Fig. 26. Accumulation fails to predict efficacy of therapeutic nanoparticle PDPA-PTX....30

Supplementary Fig. 27. Prediction of 4T1 tumour response to doxorubicin liposome using BiRN technology....31

2. Supplementary Tables....32

Supplementary Table 1. Characterization of PEG_{114}-*b*-$PDPA_{80}$-Dye_n copolymers and the resulting nanoprobes. 32

Supplementary Table 2. Characterization of different BiRN nanoprobes. 33

1. Supplementary Figures

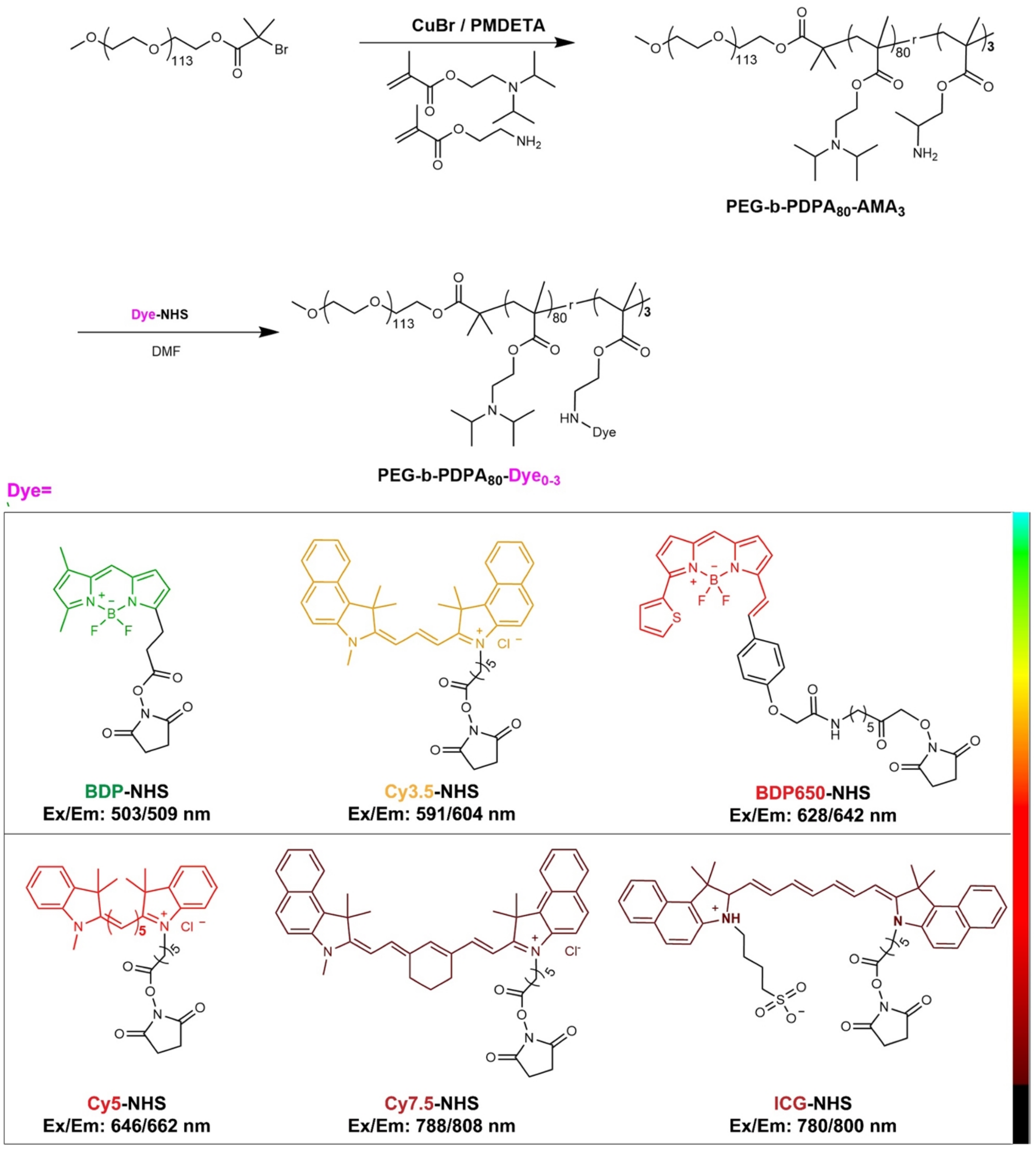

Supplementary Fig. 1. Syntheses of PEG_{114}-*b*-$PDPA_{80}$-AMA_3 and dye-conjugated PEG_{114}-*b*-$PDPA_{80}$ copolymers.

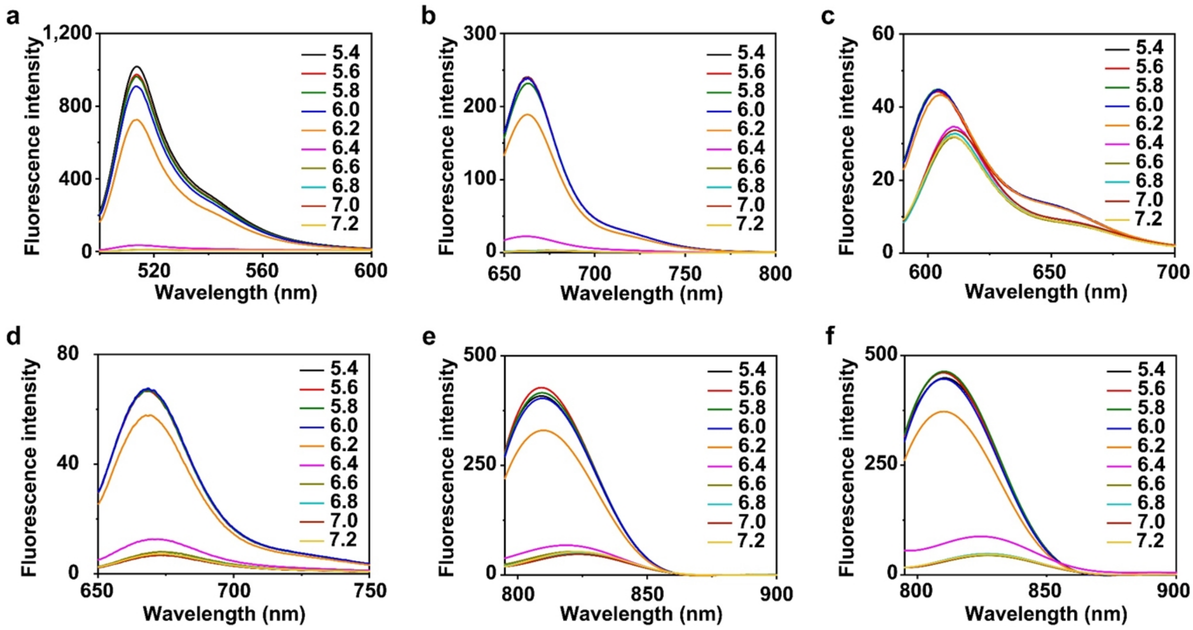

Supplementary Fig. 2. pH-dependent fluorescence emission spectra of dye-conjugated PEG_{114}-*b*-$PDPA_{80}$ copolymers: (a) PDPA-BDP, (b) PDPA-Cy5, (c) PDPA-Cy3.5, (d) PDPA-BDP650, (e) PDPA-Cy7.5 and (f) PDPA-ICG.

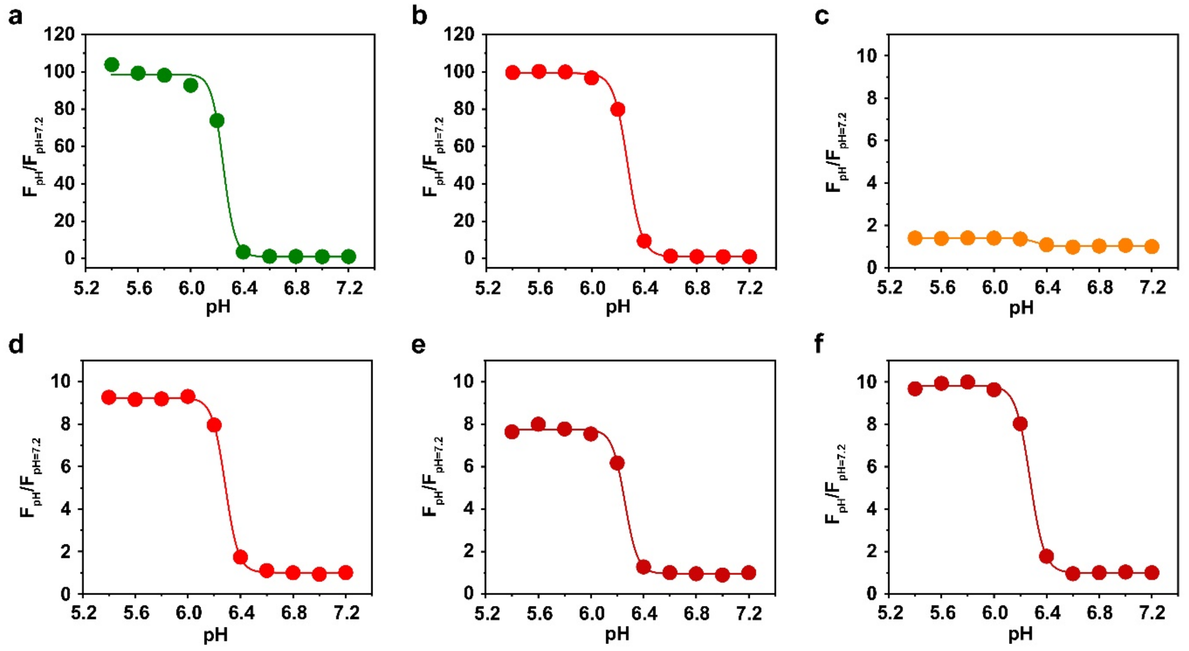

Supplementary Fig. 3. Fluorescence ratio as a function of pH for dye-conjugated PEG$_{114}$-*b*-PDPA$_{80}$ copolymers: (a) PDPA-BDP, (b) PDPA-Cy5, (c) PDPA-Cy3.5, (d) PDPA-BDP650, (e) PDPA-Cy7.5 and (f) PDPA-ICG.

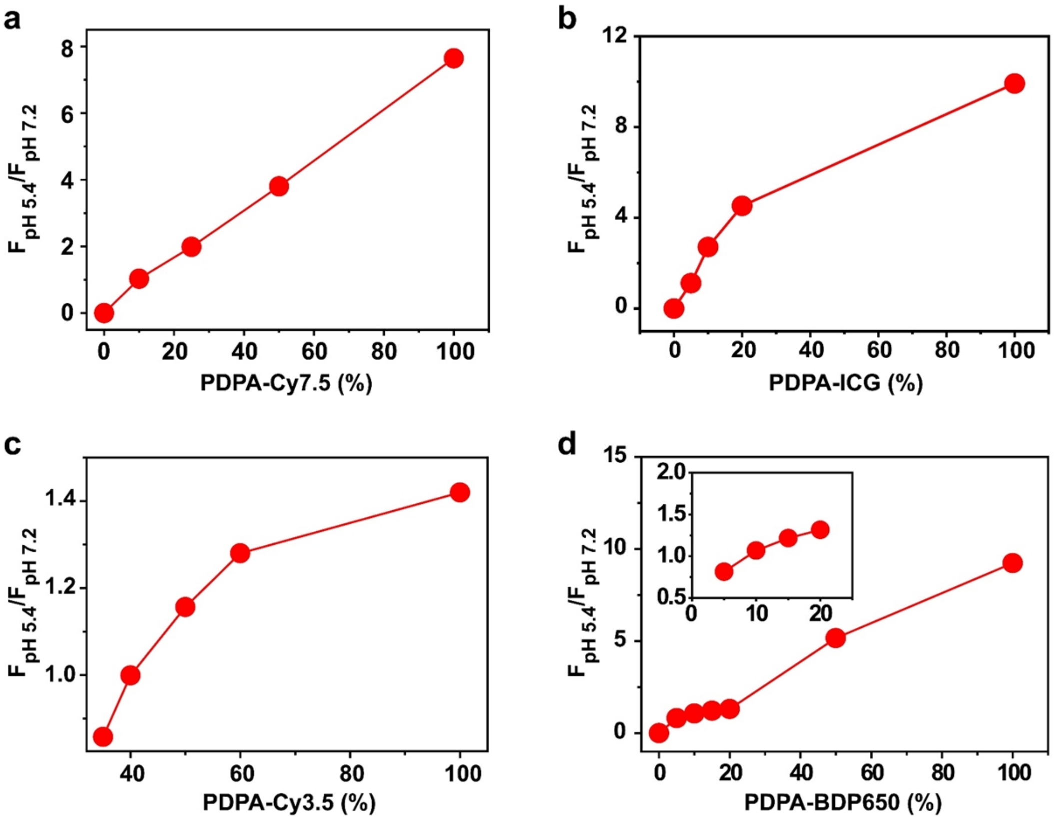

Supplementary Fig. 4. Characterization of always-ON modules for rational design of binary ratiometric nanoreporters. Fluorescence activation ratio ($F_{pH\ 5.4}/F_{pH\ 7.2}$) of potential 'always-ON' probes as a function of molar ratio of **(a)** PDPA-$Cy7.5_1$, **(b)** PDPA-$ICG_{0.5}$, **(c)** PDPA-$Cy3.5_1$, and **(d)** PDPA-$BDP650_1$ over the total polymers. The inset in **d** showed the fluorescence intensity ratio was ~1.0 with 10% molar ratio of PDPA-BDP650 polymers. The polymer concentration is 100 μg mL^{-1}.

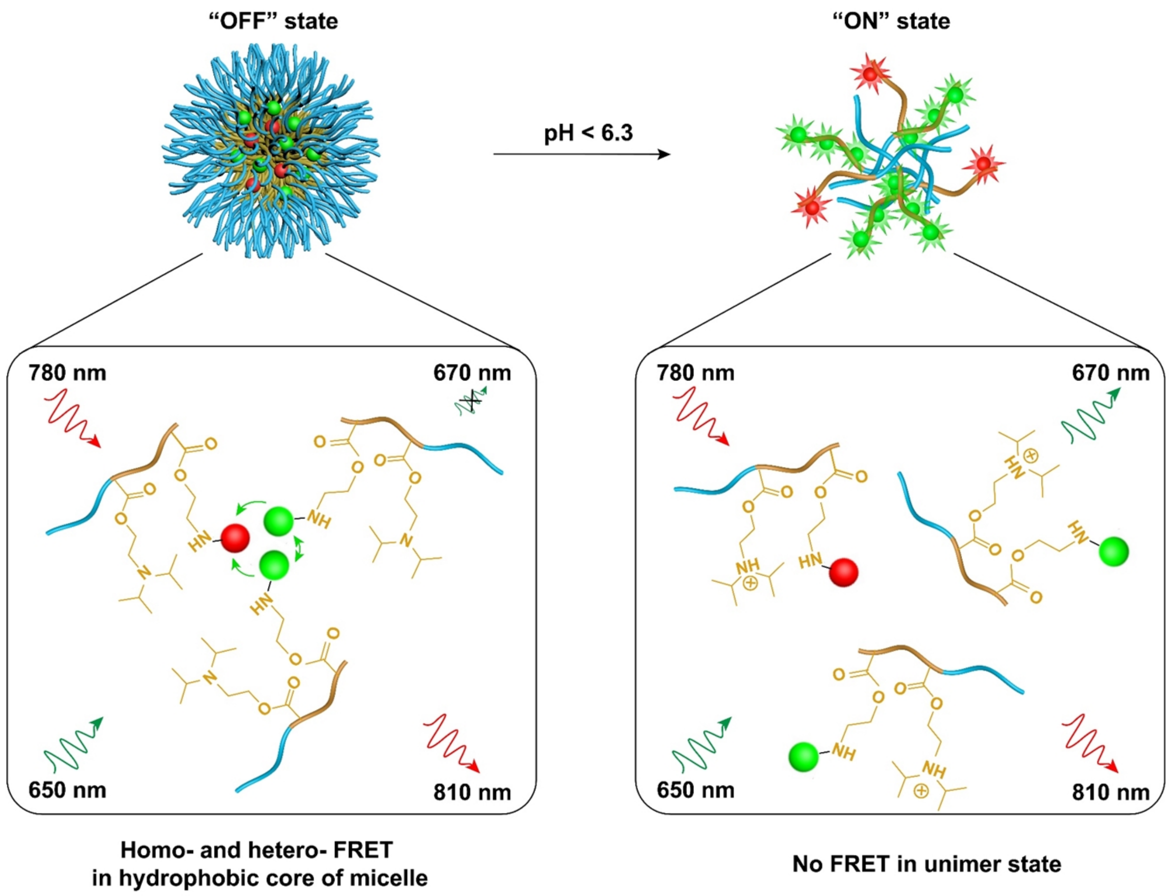

Supplementary Fig. 5. The "turn-on" mechanism of BiRN probe. At pH > 6.3, the hydrophobic segments of PEG-*b*-P(DPA-*r*-Dye) polymer self-assemble into the micelle cores, leading to fluorescence quenching of OFF-ON module by homo- and hetero-FRET (left panel). At pH < 6.3, protonation of the P(DPA-*r*-Dye) segments results in micelle dissociation with dramatic enhancement in fluorescent signal due to the abolish of FRET effect between dye molecules (right panel). In the whole process, the always-ON module presents a constant fluorescence signal.

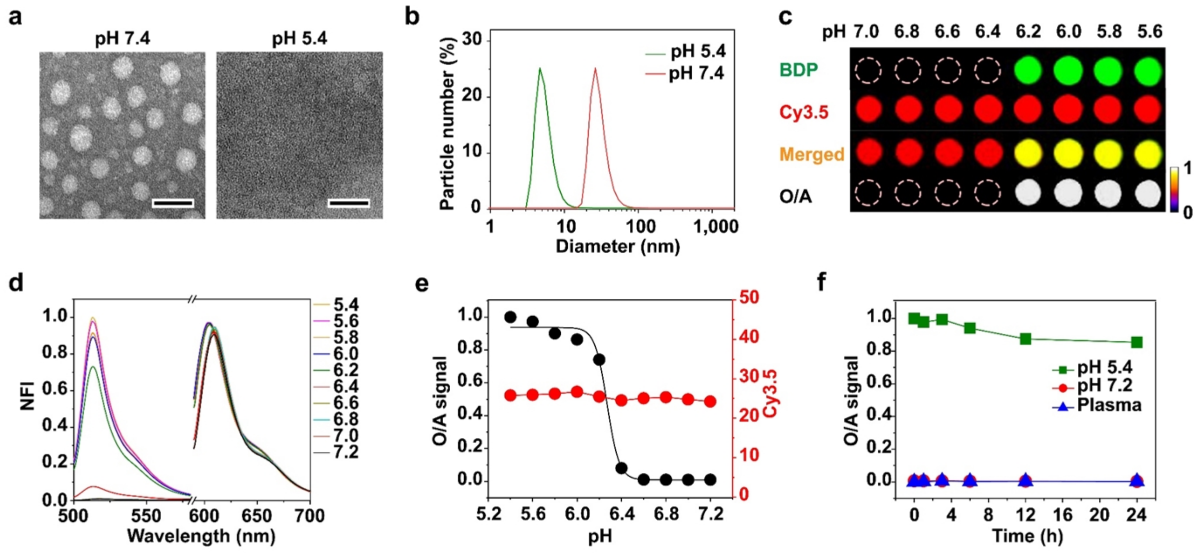

Supplementary Fig. 6. Characterization of the binary ratiometric nanoreporter in visible window. (a) Representative TEM images of micelle at pH 7.4 and 5.4. Concentration of polymer, 1 mg mL^{-1}; scale bar, 50 nm. **(b)** Particle size distribution at pH 7.4 and 5.4 measured by dynamic light scattering. Polymer concentration, 1 mg mL^{-1}. **(c)** Fluorescent images of $BiRN_{Vis}$ solutions at different pH in 384-well plate. The single channel images were obtained by in vivo imaging system ($\lambda_{ex}/\lambda_{em}$: 500/540 nm for BDP, and 570/620 nm for Cy3.5). The merged and pseudo-coloured ratio images were generated using ImageJ software. **(d)** The $BiRN_{Vis}$ in various pH solutions was excited at 488 nm and 575 nm, respectively. The emission spectra were collected separately. **(e)** Fluorescence intensity ratio of BDP to Cy3.5 and fluorescence intensity of Cy3.5 (red) as a function of pH for $BiRN_{Vis}$. **(f)** The signal ratios of OFF-ON to always-ON modules remain stable in fresh mouse plasma and PBS buffer over 24 h at 37 °C. The polymer concentration is 100 μg mL^{-1}.

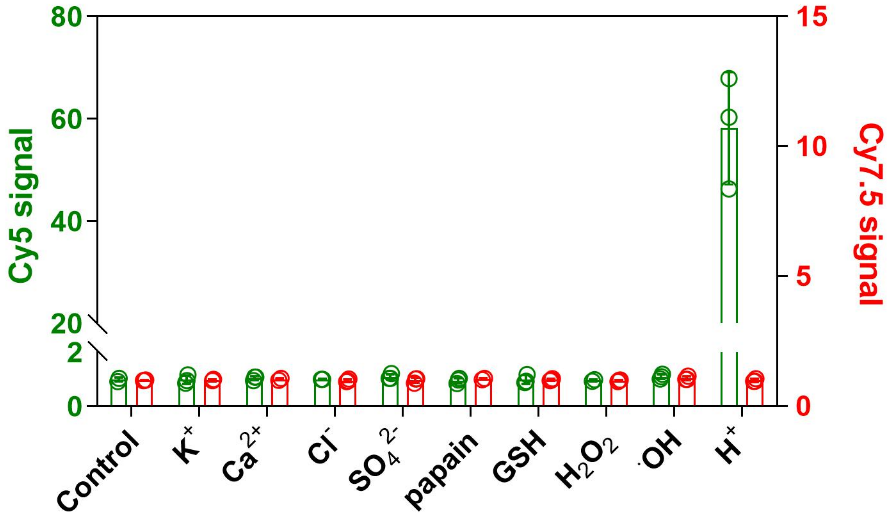

Supplementary Fig. 7. The fluorescence stability of BiRN in the presence of ions, GSH, protease or ROS. The fluorescence signals of Cy5 and Cy7.5 in the presence of K^+ (140 mM), Ca^{2+} (5 mM), Cl^- (114 mM), SO_4^{2-} (20 mM) ions, GSH (10 mM), papain (5 μM), H_2O_2 (1 μM) and •OH (1 μM). BiRN concentration, 0.1 mg mL^{-1}. Data are presented as mean ± s.d. (n = 3 biologically independent samples).

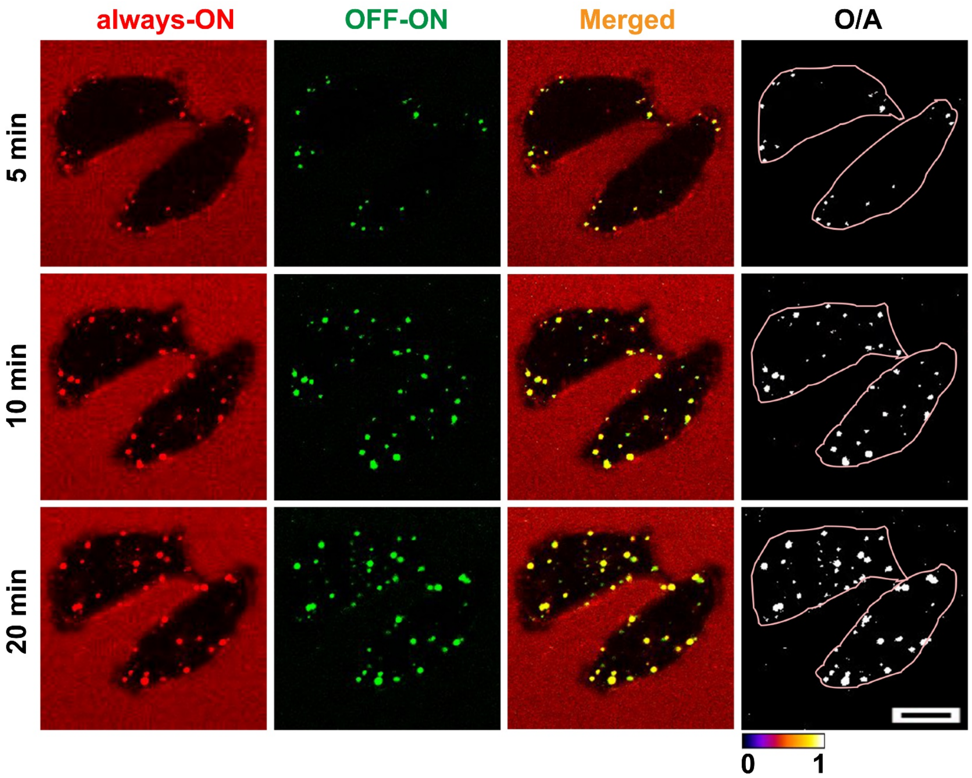

Supplementary Fig. 8. Confocal images of A549 cells treated with BiRN at the indicated incubation time. Ratiometric images only present the internalized nanoprobe, which were generated from the ratio of BDP to Cy3.5 by ImageJ software. Pink lines distinguish intracellular regions from extracellular culture medium. Concentration of $BiRN_{Vis}$ is 100 µg mL^{-1}. Red, Cy3.5; green, BDP; yellow, colocalization of Cy3.5 and BDP FL; white, the ratiometric O/A signal. Scale bar, 20 µm.

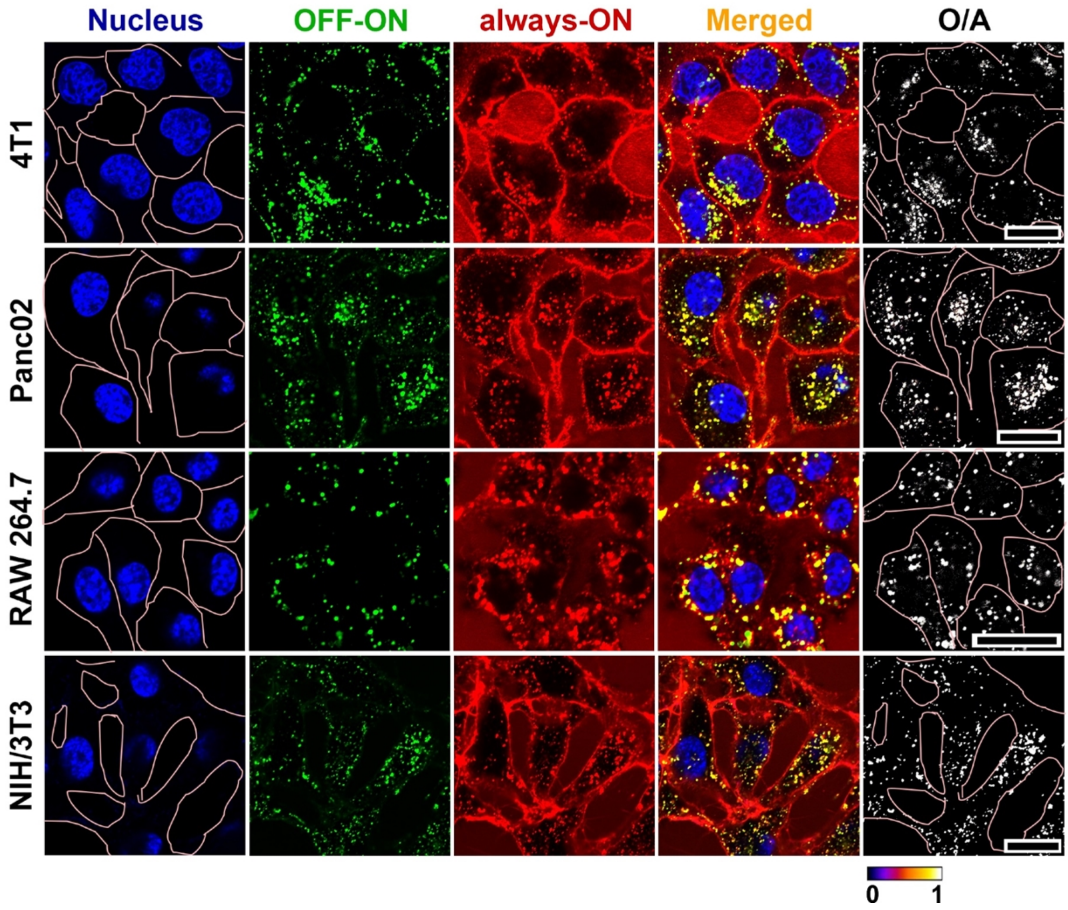

Supplementary Fig. 9. Confocal images of representative cells treated with BiRN for 0.5 h. The cell lines include cancer cells (4T1 breast cancer cell and Panc02 pancreatic cancer) and normal cells (RAW 264.7 macrophage and NIH/3T3 fibroblast). Yellow colour in the merged channels indicates colocalization of OFF-ON module with always-ON module. Bright spots in ratio channels indicate nanoprobes internalized by the cells. Pink lines distinguish intracellular regions from extracellular culture medium. The extracellular distributed and cell surface-bound nanoparticles were successfully excluded for internalization evaluation by ratiometric imaging. Concentration of $BiRN_{Vis}$ is 100 μg mL^{-1}. Scale bar, 20 μm.

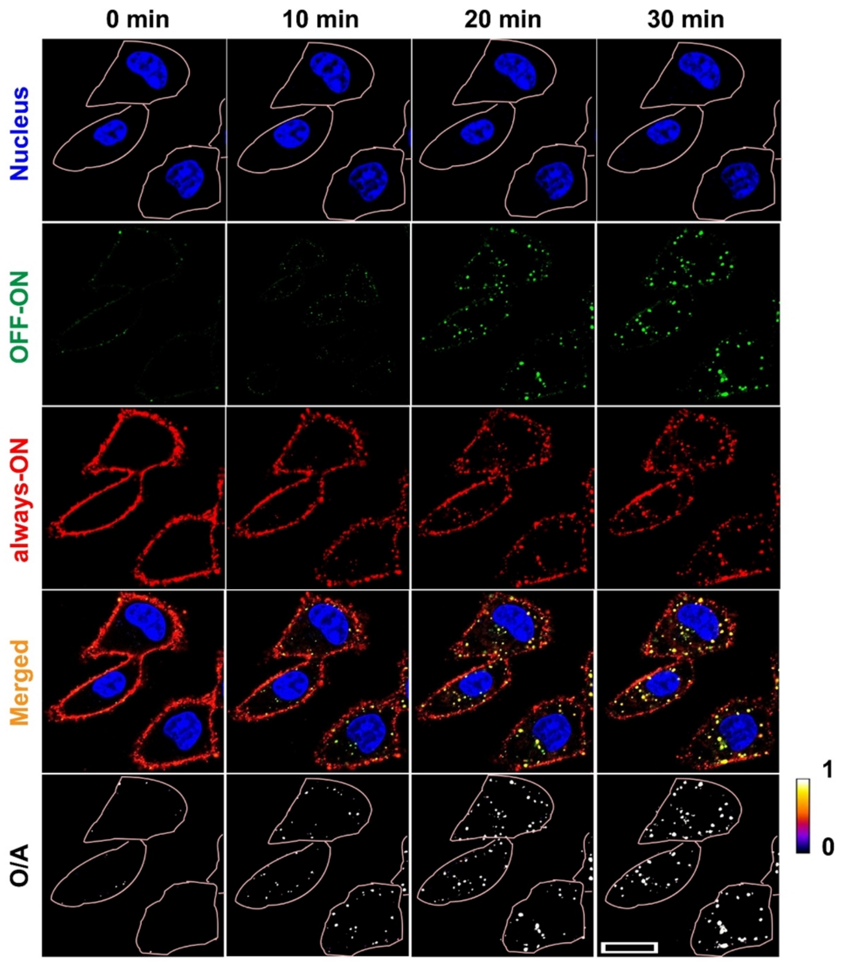

Supplementary Fig. 10. Kinetics study of BiRN internalization in A549 lung cancer cells. Confocal images of A549 cells pulsed with $BiRN_{Vis}$ (100 µg mL^{-1}) at the indicated chase time. The cell surface-bound nanoparticles were successfully excluded for internalization monitoring by ratiometric imaging. Scale bar, 20 µm.

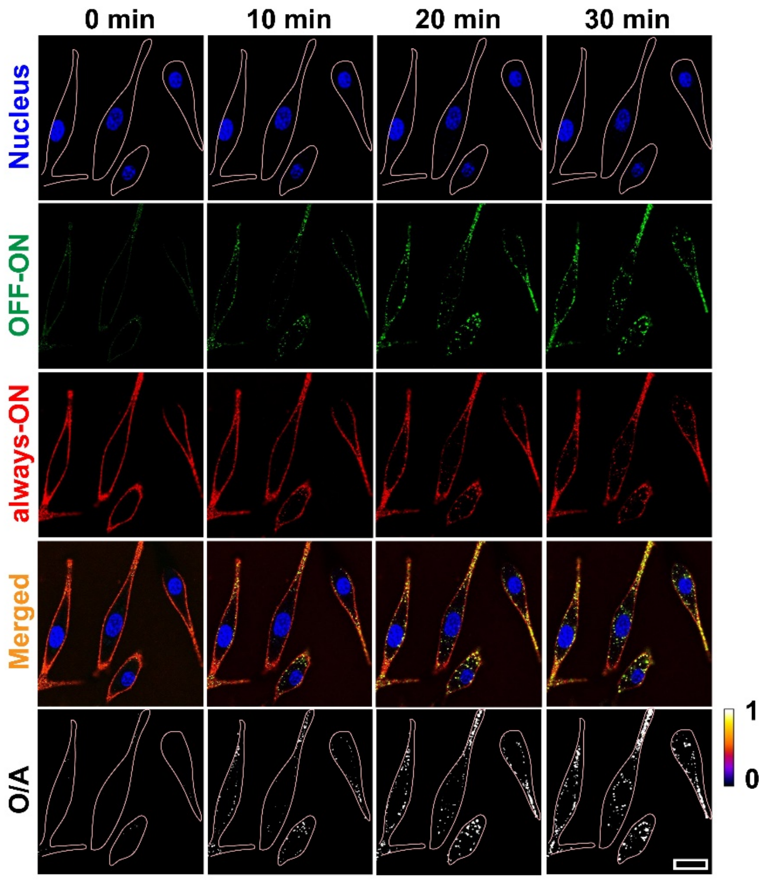

Supplementary Fig. 11. Kinetics study of BiRN internalization in Panc02 pancreatic cancer cells. Confocal images of Panc02 cells pulsed with $BiRN_{Vis}$ at the indicated chase time. Concentration of $BiRN_{Vis}$ is 100 µg mL^{-1}. Scale bar, 20 µm.

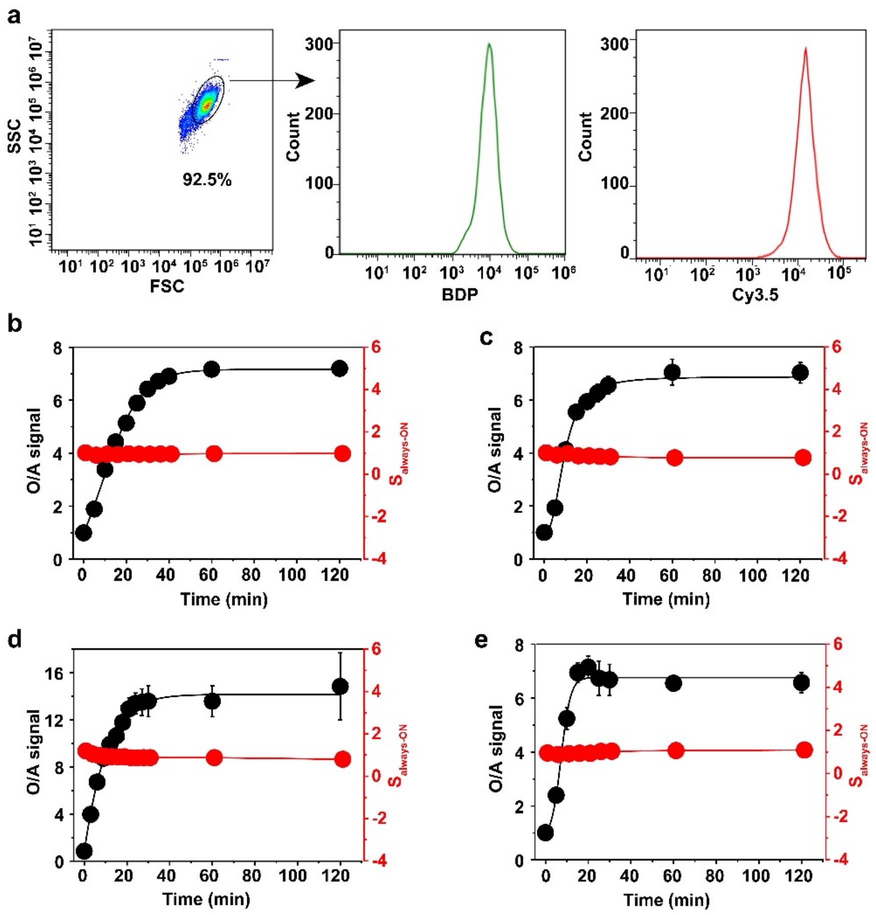

Supplementary Fig. 12. Kinetics study of BiRN internalization in various cells. (a) Cell populations were gated for a live population using FSC and SSC plot of cell sample. The gate was set to remove cell debris (small FSC *v* SSC) and large aggregates of cells (large FSC or SSC) and used across all samples. This live population was then used in fluorescent histograms. Cy3.5 fluorescence quantitative analysis (red) and quantitative O/A signal analysis (black) of **(b)** 4T1, **(c)** BxPC-3, **(d)** Panc02 and **(e)** MCF-7 cells pulsed with $BiRN_{Vis}$ at the indicated chase time by flow cytometry analyses. Concentration of $BiRN_{Vis}$ is 100 μg mL^{-1}. Data are presented as mean ± s.d. (n = 3 biologically independent experiments).

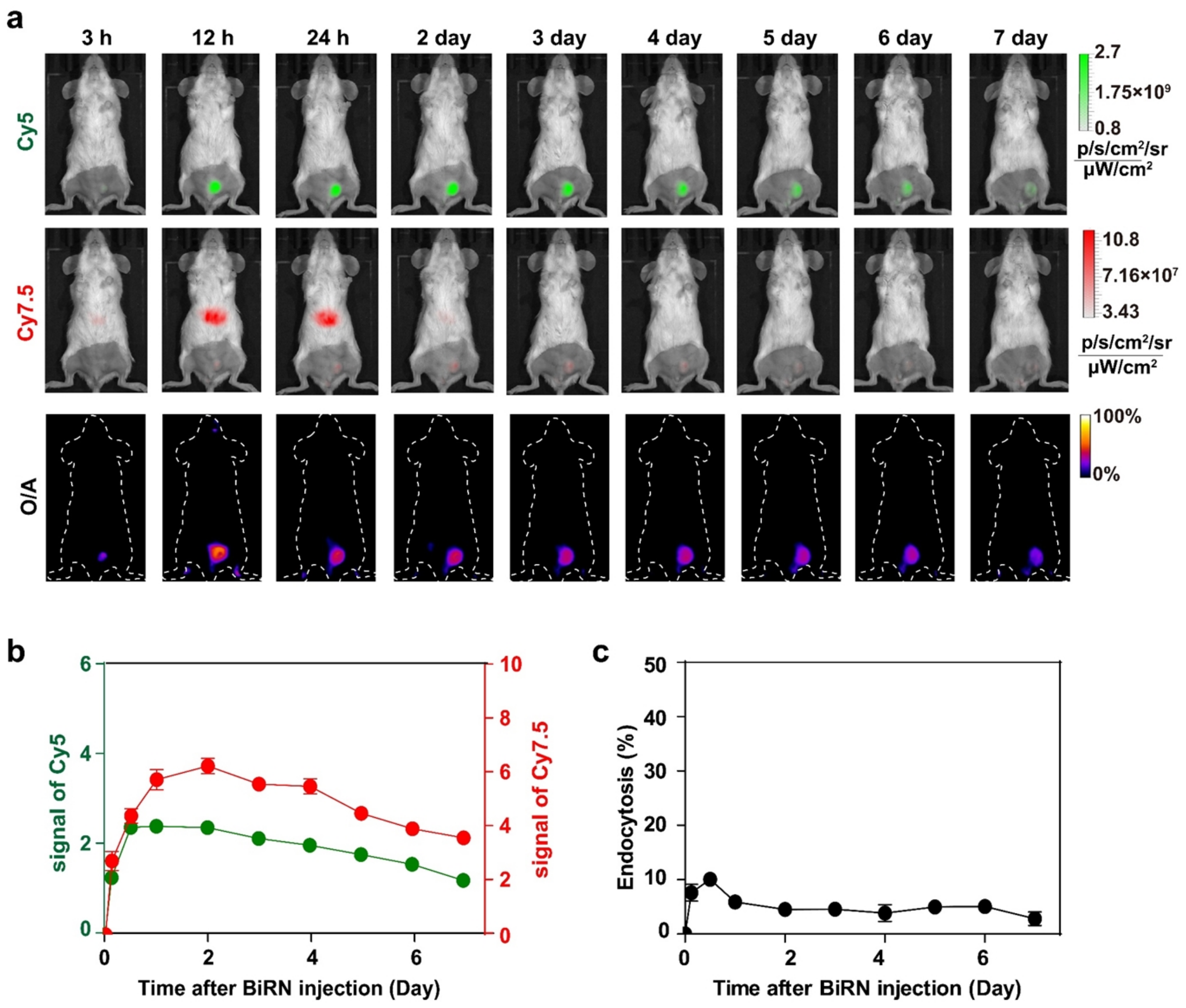

Supplementary Fig. 13. Long-term monitoring of BiRN internalization in vivo. (a) 4T1 tumour-bearing mice were administrated intravenously with BiRN (20 mg kg^{-1}), dual-channel fluorescence images were captured at selected time-points. O/A channel images were generated from the ratio of Cy5 to Cy7.5 by ImageJ software. **(b)** Time-dependent Cy5 and Cy7.5 fluorescence intensity after BiRN injection. **(c)** Time-dependent endocytosis percentage of BiRN in tumour after nanoparticle injection. Data were presented as mean ± s.d. (n = 5 biologically independent mice).

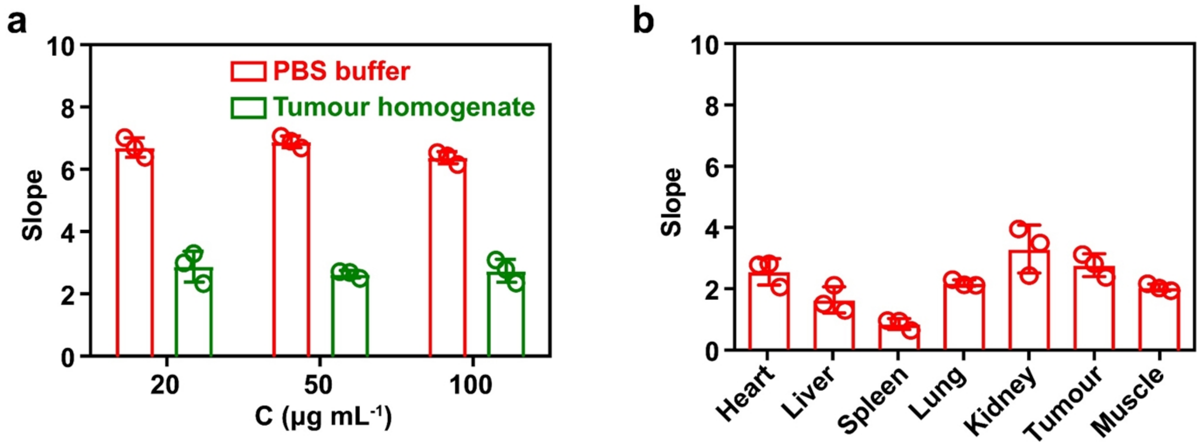

Supplementary Fig. 14. Establishment of quantitative analysis method for BiRN internalization. (a) $BiRN_{NIR}$ diluted in PBS and tumour homogenates (n = 3 biologically independent experiments) with varying concentrations (20, 50, 100 μg mL^{-1}) showed a concentration-independent pattern for the slope of calibration curves. **(b)** Slopes of calibration curves established by diluting $BiRN_{NIR}$ to different tissue homogenates, including heart, liver, spleen, lung, kidney, tumour, and muscle. Experiments were performed with 100 μg mL^{-1} $BiRN_{NIR}$. Data are presented as mean ± s.d. (n = 3 biologically independent experiments).

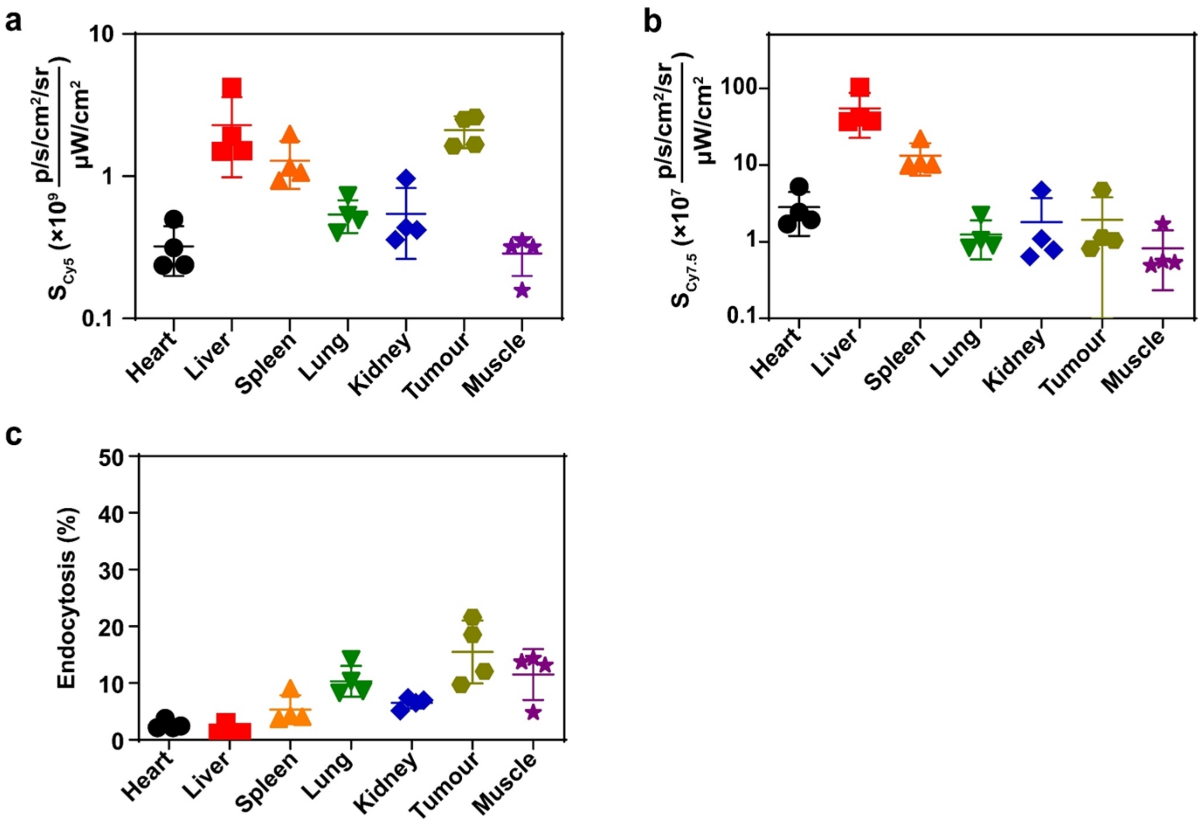

Supplementary Fig. 15. Quantification of the nanoparticle accumulation and endocytosis percentage in different organs. At 24 h post-injection of $BiRN_{NIR}$, 4T1 breast tumour-bearing mice were euthanized and dissected organs were flurescently imaged. **(a)** Cy5 and **(b)** Cy7.5 fluorescence signals in different dissected organs. **(c)** Endocytosis percentage of BiRN in different dissected organs. Data are presented as mean ± s.d. (n = 4 biologically independent mice).

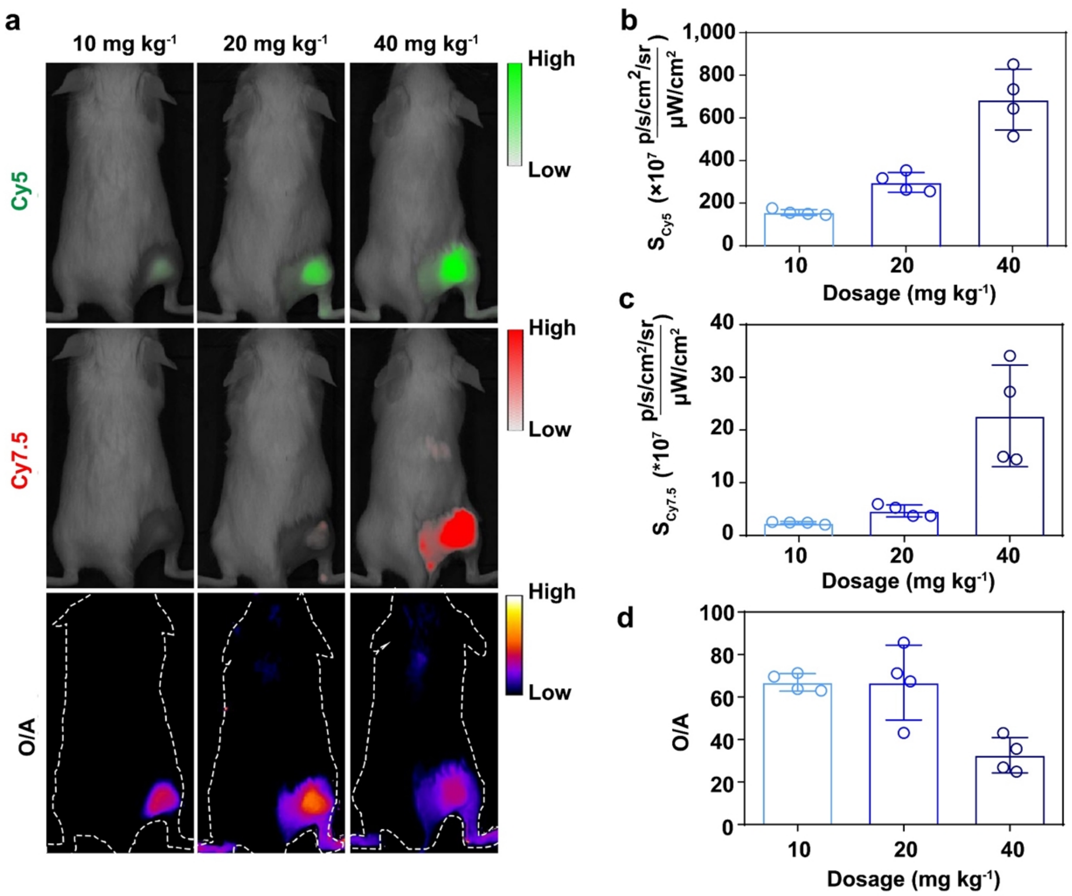

Supplementary Fig. 16. Study on the concentration-dependence of accumulation and endocytosis in vivo. (a) NIR fluorescence images of 4T1 tumour-bearing mice were captured at 24 h post-injection of different doses of BiRN. (**b**) Fluorescence intensity of Cy5 signals from the excised tumours at 24 h post-injection of different doses of $BiRN_{NIR}$ ($n = 4$ biologically independent mice). (**c**) Fluorescence intensity of Cy7.5 signals from the excised tumours at 24 h post-injection of different doses of $BiRN_{NIR}$. **(d)** Ratiometric O/A signal indicates endocytosis percentage of tumour with BiRN at different dosage. Data are presented as mean ± s.d. ($n = 4$ biologically independent mice).

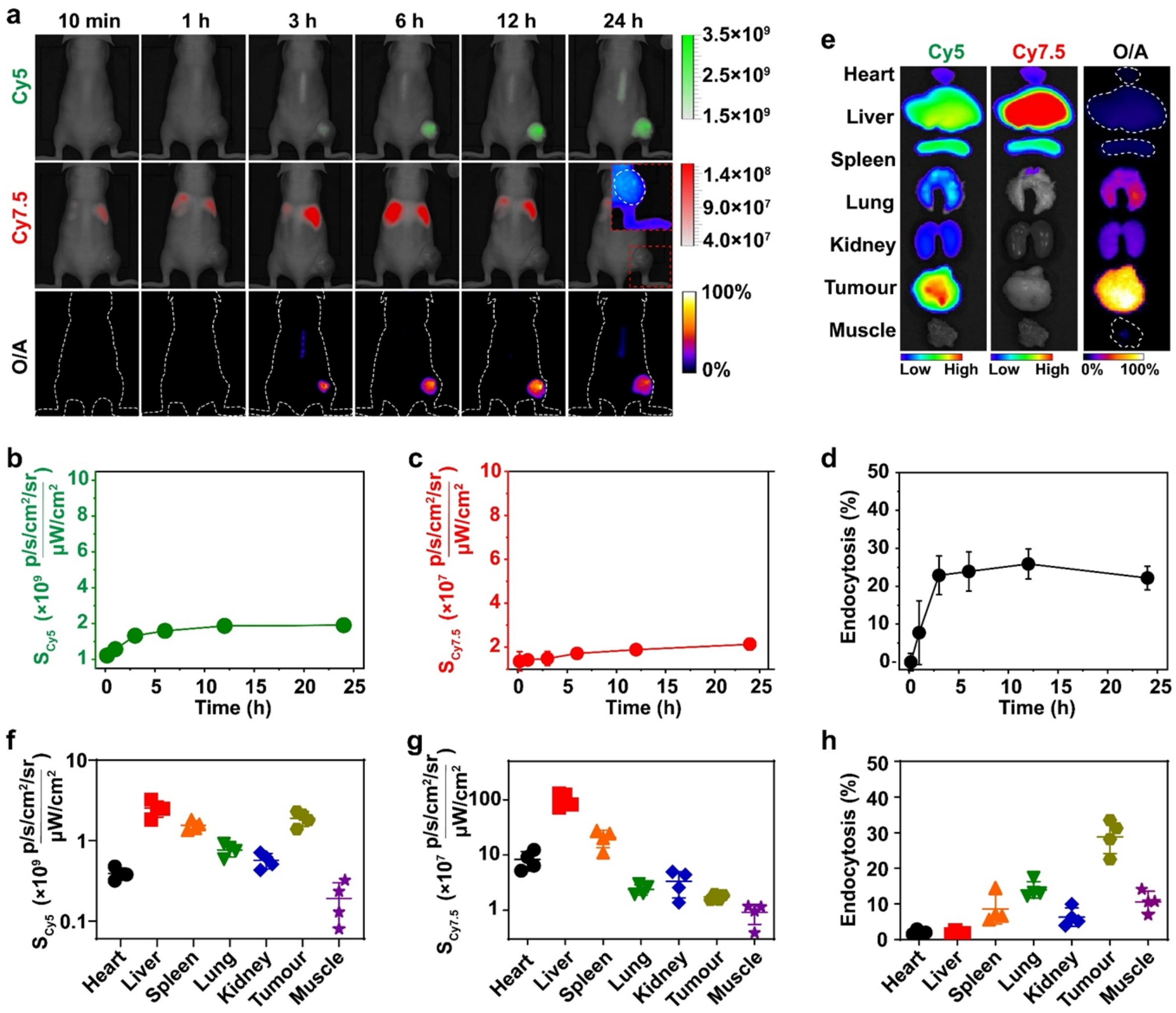

Supplementary Fig. 17. Real-time monitoring of tumour accumulation and cellular endocytosis of BiRN in MCF-7 breast tumour xenografts. (a) MCF-7 tumour-bearing mice were injected with $BiRN_{NIR}$ nanoprobe at 20 mg kg^{-1}, dual-channel fluorescence images at pre-determined time points were captured. The inset in **a** was heat map of Cy7.5 fluorescence signal in magnified scale bar and the tumour area was marked with white dash line. **(b)** Cy5 fluorescence intensity (endocytosis amount), **(c)** Cy7.5 fluorescence intensity (accumulation), and **(d)** endocytosis percentage as a function of time after $BiRN_{NIR}$ injection. **(e)** At 24 h post-injection, mice were euthanized and the dissected organs were visualized. **(f)** Cy5 fluorescence signals, **(g)** Cy7.5 fluorescence signals and **(h)** endocytosis percentage of the dissected organs. Data are presented as mean ± s.d. (n = 4 biologically independent mice).

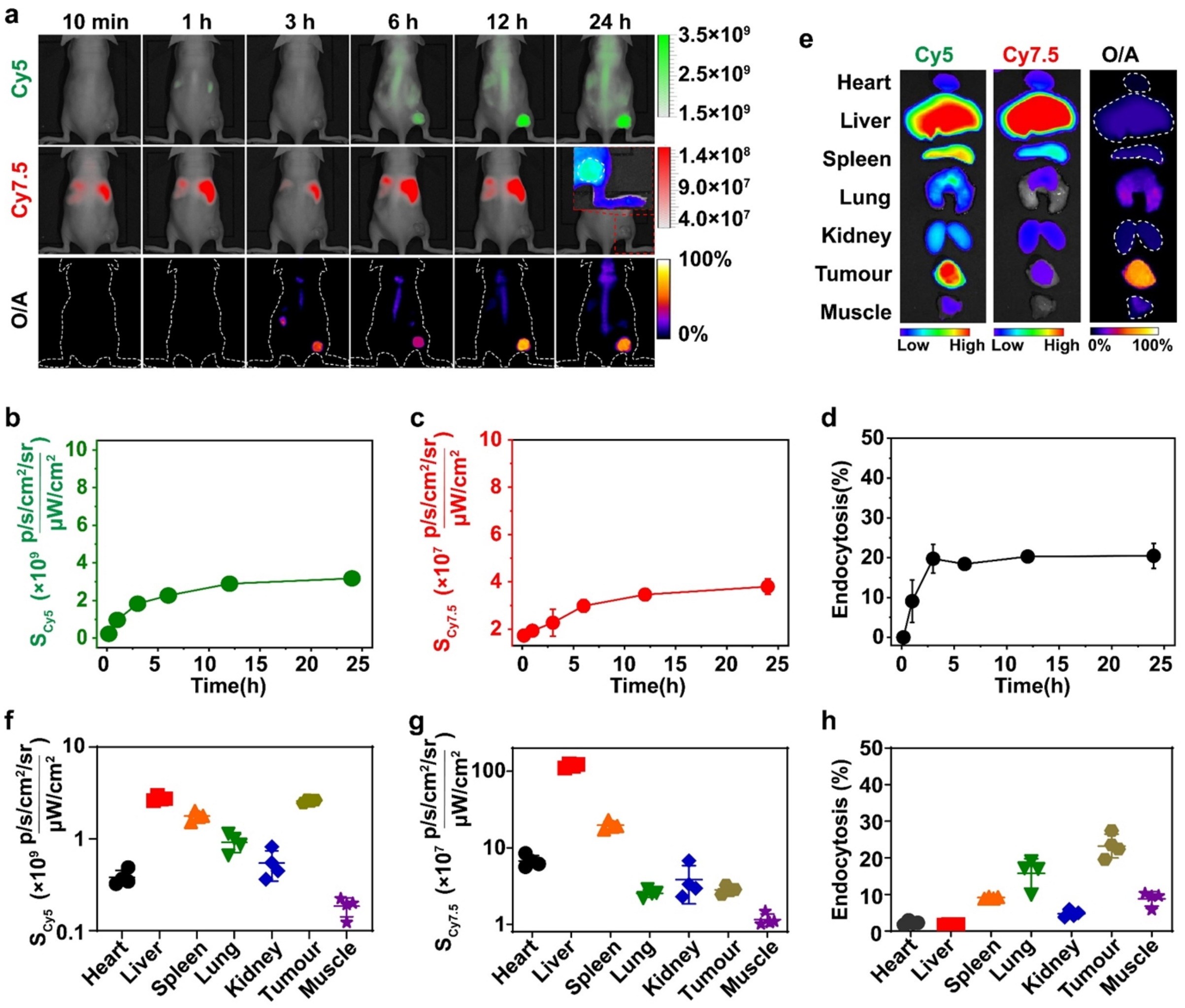

Supplementary Fig. 18. Real-time monitoring of tumour accumulation and cellular endocytosis of BiRN in Panc02 pancreatic tumour xenografts. (a) Panc02 tumour-bearing mice were injected with $BiRN_{NIR}$ nanoprobe at 20 mg kg^{-1}, dual-channel fluorescence images at pre-determined time points were captured. The inset in **a** was heat map of Cy7.5 fluorescence signal in magnified scale bar and the tumour area was marked with white dash line. **(b)** Cy5 fluorescence intensity (endocytosis amount), **(c)** Cy7.5 fluorescence intensity (accumulation), and **(d)** endocytosis percentage as a function of time after $BiRN_{NIR}$ injection. **(e)** At 24 h post-injection, mice were euthanized and the dissected organs were visualized. **(f)** Cy5 fluorescence signals, **(g)** Cy7.5 fluorescence signals and **(h)** endocytosis percentage of the dissected organs. Data are presented as mean ± s.d. (*n* = 4 biologically independent mice).

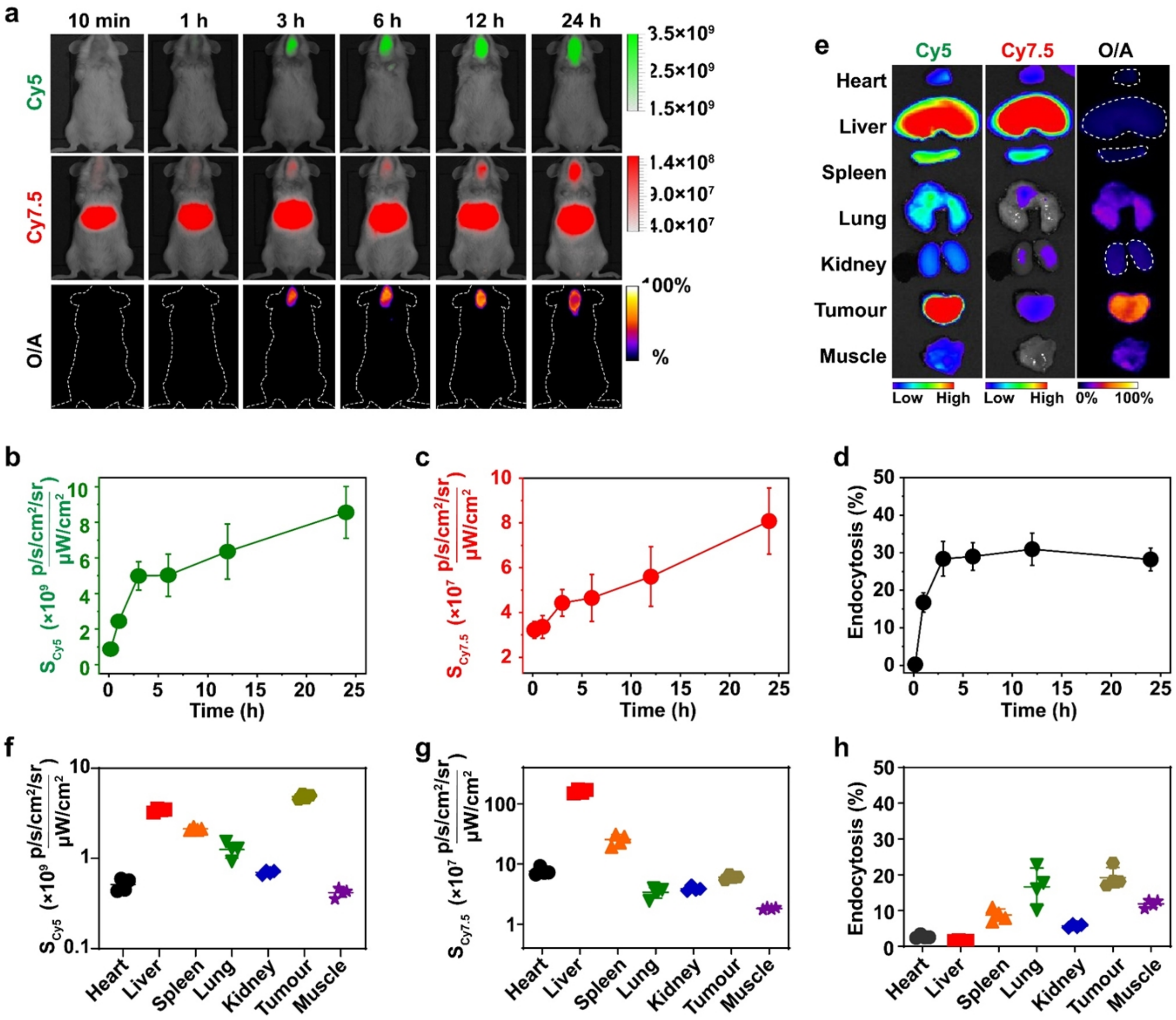

Supplementary Fig. 19. Real-time monitoring of tumour accumulation and cellular endocytosis of BiRN in HN5 head & neck tumour xenografts. (a) HN5 tumour-bearing mice were injected with $BiRN_{NIR}$ nanoprobe at 20 mg kg^{-1}, dual-channel fluorescence images at pre-determined time points were captured. The inset in **a** was heat map of Cy7.5 fluorescence signal in magnified scale bar and the tumour area was marked with white dash line. **(b)** Cy5 fluorescence intensity (endocytosis amount), **(c)** Cy7.5 fluorescence intensity (accumulation), and **(d)** endocytosis percentage as a function of time after $BiRN_{NIR}$ injection. **(e)** At 24 h post-injection, mice were euthanized and the dissected organs were visualized. **(f)** Cy5 fluorescence signals, **(g)** Cy7.5 fluorescence signals and **(h)** endocytosis percentage of the dissected organs. Data are presented as mean ± s.d. (n = 4 biologically independent mice).

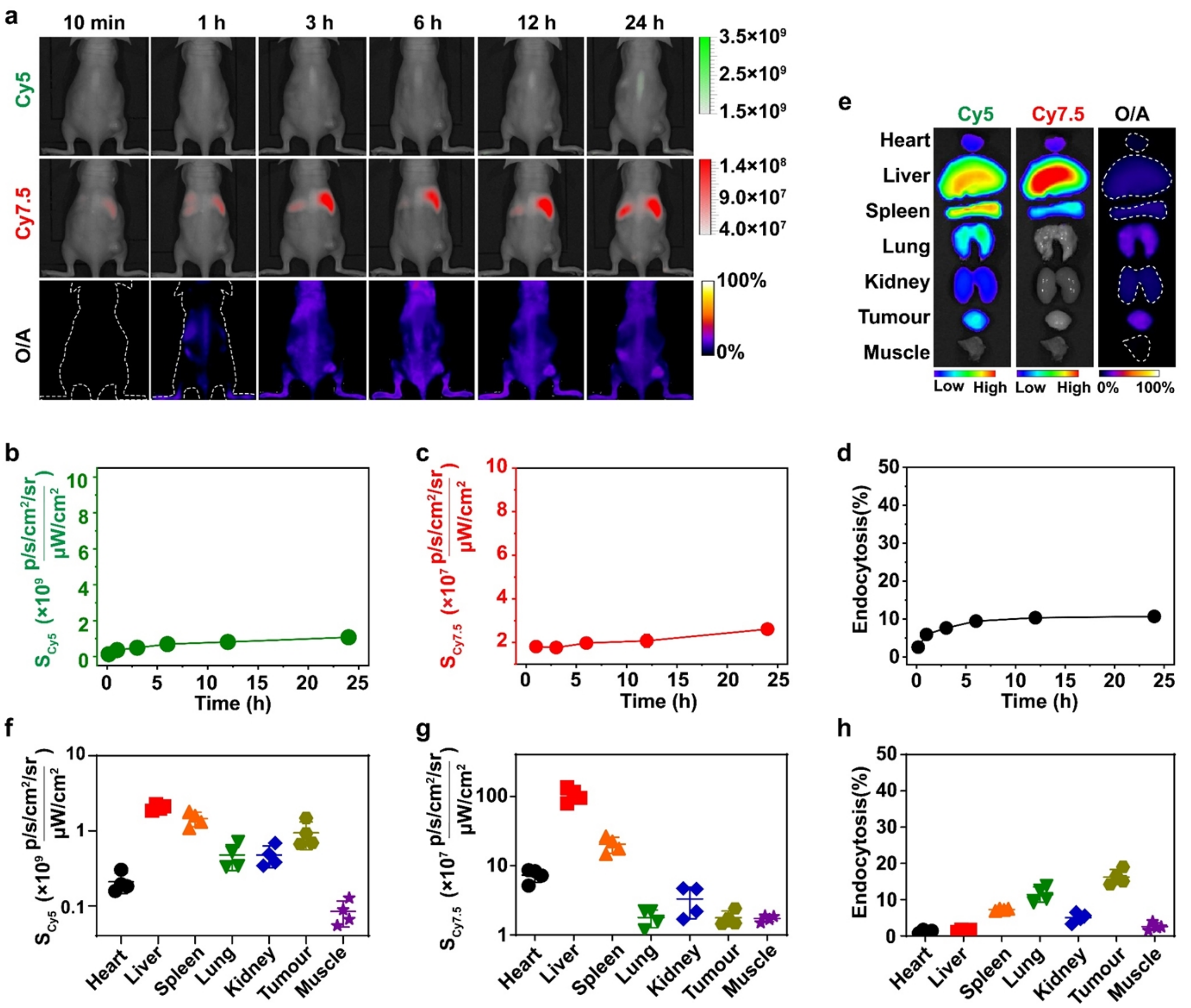

Supplementary Fig. 20. Real-time monitoring of tumour accumulation and cellular endocytosis of BiRN in A549 lung cancer xenografts. (a) A549 tumour-bearing mice were injected with $BiRN_{NIR}$ nanoprobe at 20 mg kg^{-1}, dual-channel fluorescence images at pre-determined time points were captured. The inset in **a** was heat map of Cy7.5 fluorescence signal in magnified scale bar and the tumour area was marked with white dash line. **(b)** Cy5 fluorescence intensity (endocytosis amount), **(c)** Cy7.5 fluorescence intensity (accumulation), and **(d)** endocytosis percentage as a function of time after $BiRN_{NIR}$ injection. **(e)** At 24 h post-injection, mice were euthanized and the dissected organs were visualized. **(f)** Cy5 fluorescence signals, **(g)** Cy7.5 fluorescence signals and **(h)** endocytosis percentage of the dissected organs. Data are presented as mean ± s.d. (n = 4 biologically independent mice).

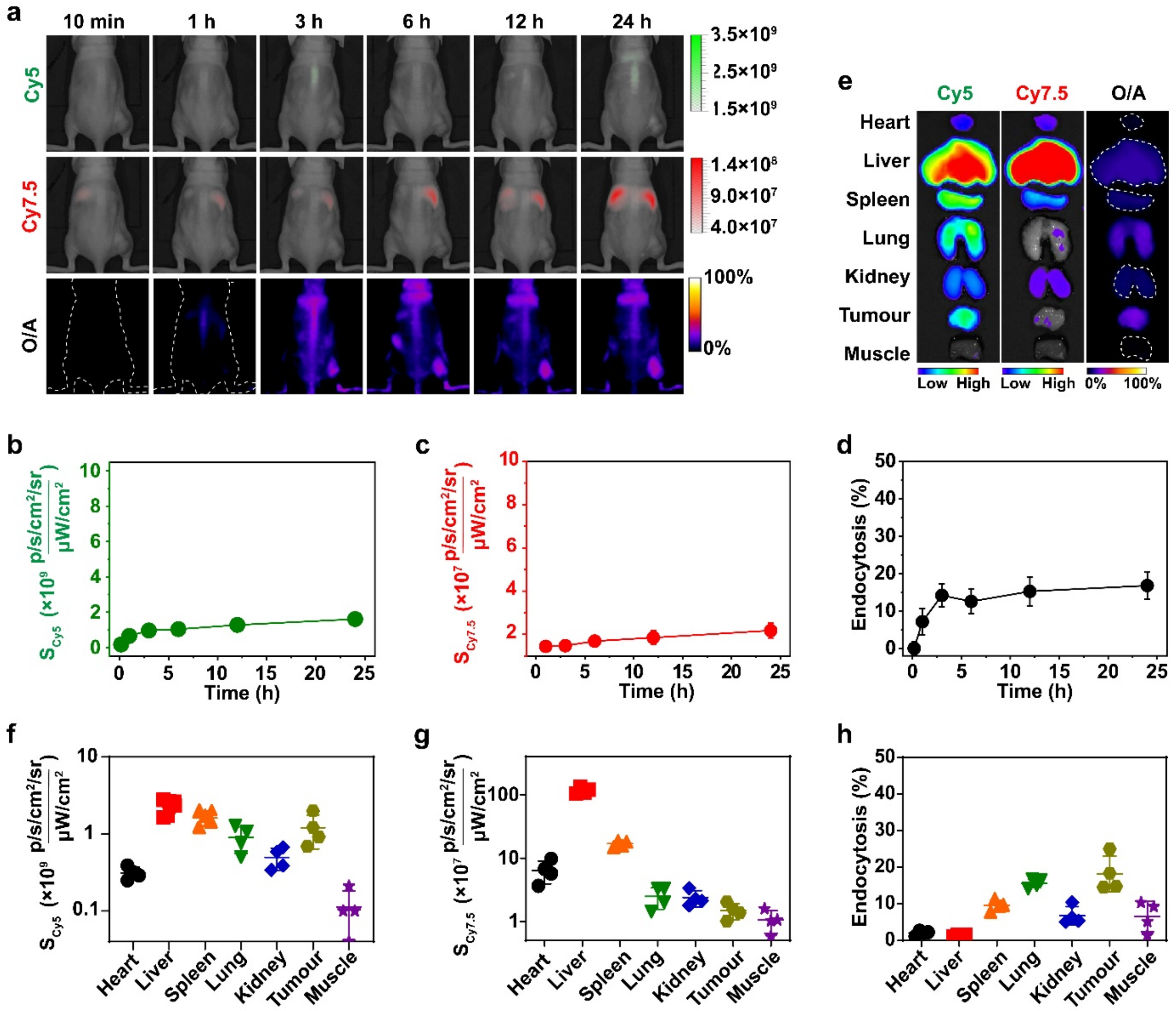

Supplementary Fig. 21. Real-time monitoring of tumour accumulation and cellular endocytosis of BiRN in BxPC-3 pancreatic tumour xenografts. (a) BxPC-3 tumour-bearing mice were injected with $BiRN_{NIR}$ nanoprobe at 20 mg kg^{-1}, dual-channel fluorescence images at pre-determined time points were captured. The inset in **a** was heat map of Cy7.5 fluorescence signal in magnified scale bar and the tumour area was marked with white dash line. **(b)** Cy5 fluorescence intensity (endocytosis amount), **(c)** Cy7.5 fluorescence intensity (accumulation), and **(d)** endocytosis percentage as a function of time after $BiRN_{NIR}$ injection. **(e)** At 24 h post-injection, mice were euthanized and the dissected organs were visualized. **(f)** Cy5 fluorescence signals, **(g)** Cy7.5 fluorescence signals and **(h)** endocytosis percentage of the dissected organs. Data are presented as mean ± s.d. (*n* = 4 biologically independent mice).

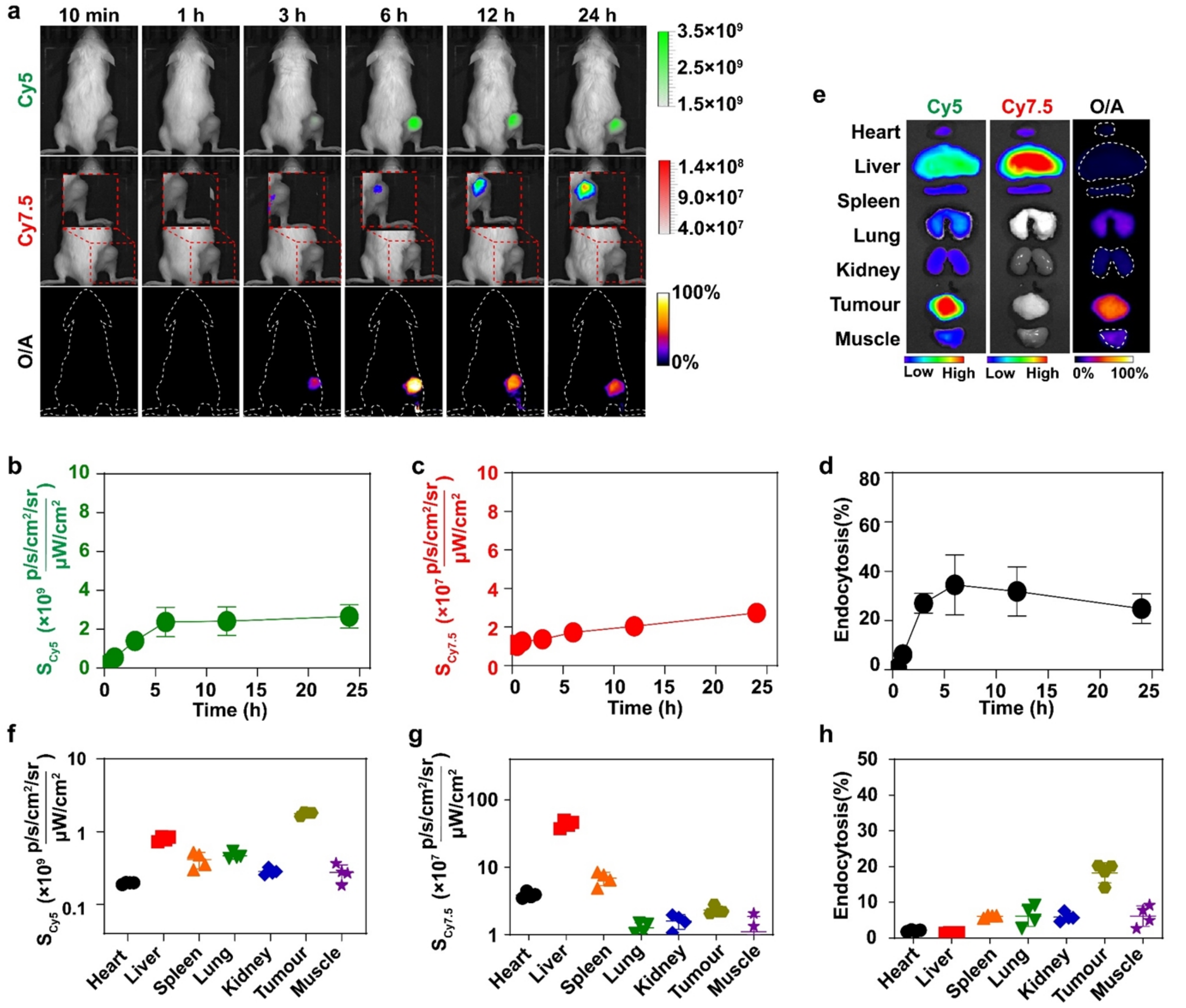

Supplementary Fig. 22. Real-time monitoring of tumour accumulation and cellular endocytosis of BiRN in PDX esophageal carcinoma model. (a) PDX tumour-bearing mice were injected with $BiRN_{NIR}$ nanoprobe at 20 mg kg^{-1}, dual-channel fluorescence images at pre-determined time points were captured. The inset in **a** was heat map of Cy7.5 fluorescence signal in magnified scale bar and the tumour area was marked with white dash line. **(b)** Cy5 fluorescence intensity (endocytosis amount), **(c)** Cy7.5 fluorescence intensity (accumulation), and **(d)** endocytosis percentage in tumor tissues as a function of time after $BiRN_{NIR}$ injection. **(e)** At 24 h post-injection, mice were euthanized and the dissected organs were visualized. **(f)** Cy5 fluorescence signals, **(g)** Cy7.5 fluorescence signals and **(h)** endocytosis percentage of the dissected organs. Data are presented as mean ± s.d. (n = 4 biologically independent mice).

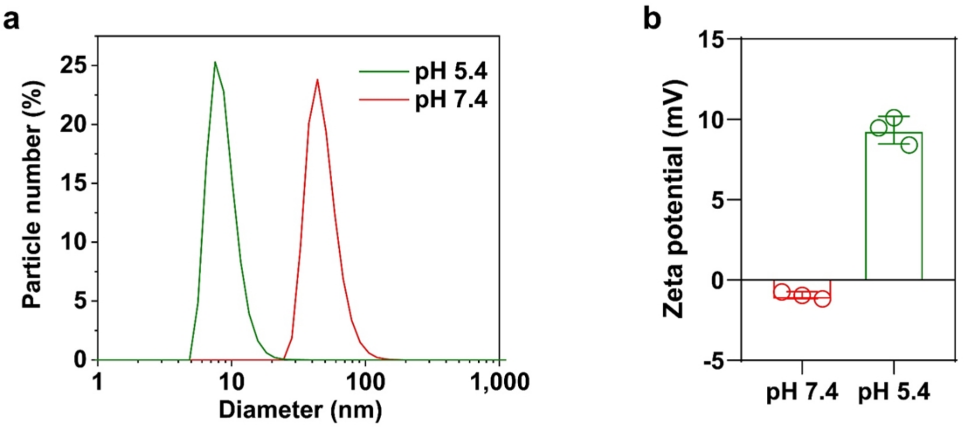

Supplementary Fig. 23. Characterization of PDPA-PTX micelles. (a) Particle size distribution and **(b)** zeta potential of PDPA-PTX micelle at pH 7.4 and 5.4 measured by dynamic light scattering (polymer concentration is 0.1 mg mL^{-1}). Data are presented as mean ± s.d. (*n* = 3 biologically independent experiments).

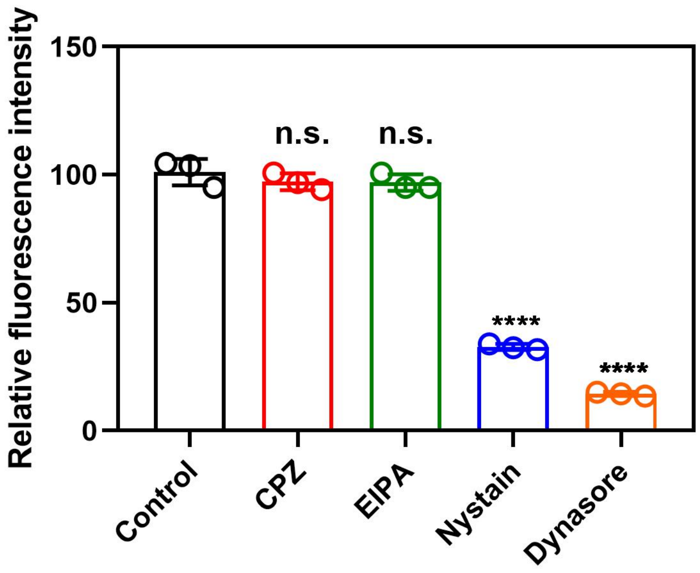

Supplementary Fig. 24. Endocytosis mechanism of PDPA-PTX micelles was evaluated in 4T1 cells. Cellular association of BiRN in the presence of different endocytosis inhibitors. Data are shown as mean ± s.d. (n = 3 biologically independent experiments). ****$P < 0.0001$, n.s., not significant, two-sided Student t-test.

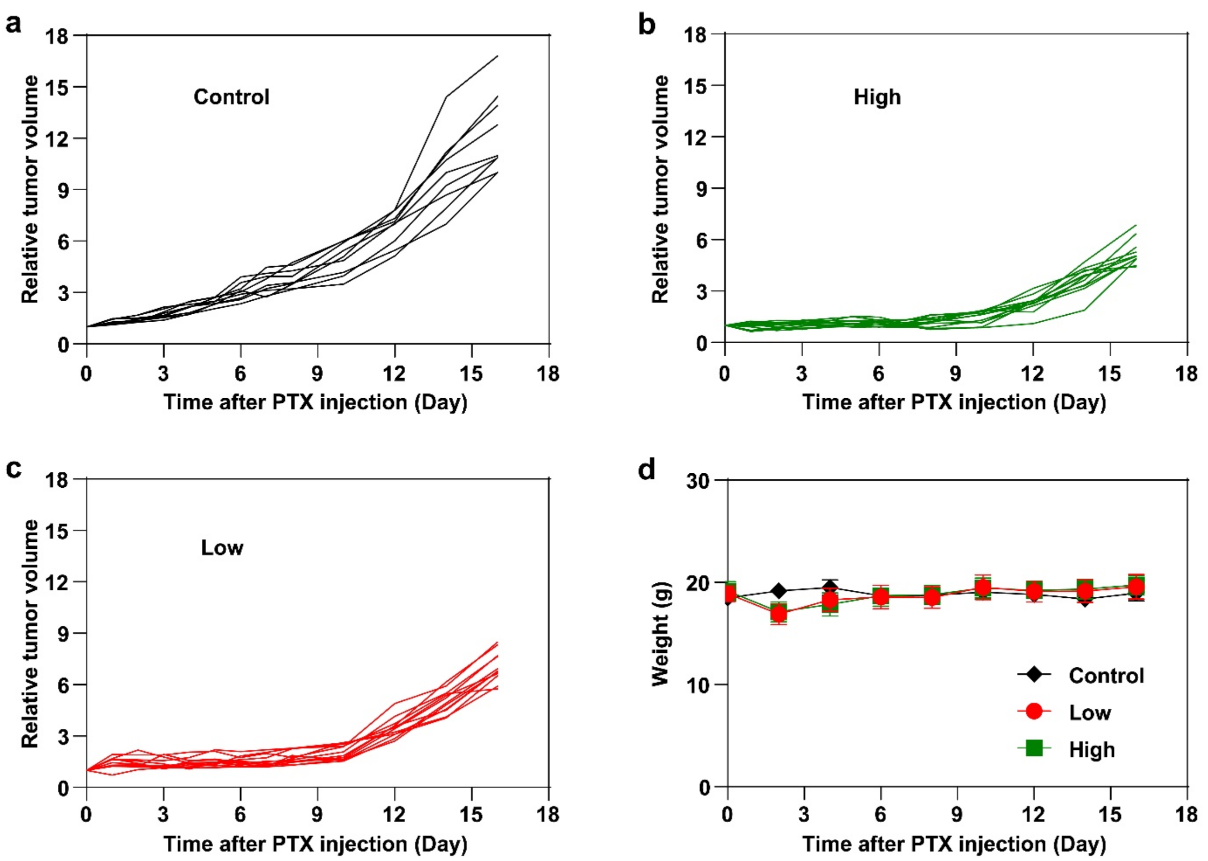

Supplementary Fig. 25. Individual tumour growth curves and weight change of 4T1 tumour-bearing mice in different groups according to Cy5 signal. (a) Individual tumour growth curves in control group (0.9% saline). (b) Individual tumour growth curves in high endocytosis group after PDPA-PTX injection. (c) Individual tumour growth curves in low endocytosis group after PDPA-PTX injection. (d) Body weight change of mice in control, high-, and low-endocytosis groups after treatments. Data are presented as mean ± s.d. (n = 8 biologically independent mice for PBS group; n = 12 biologically independent mice for other groups).

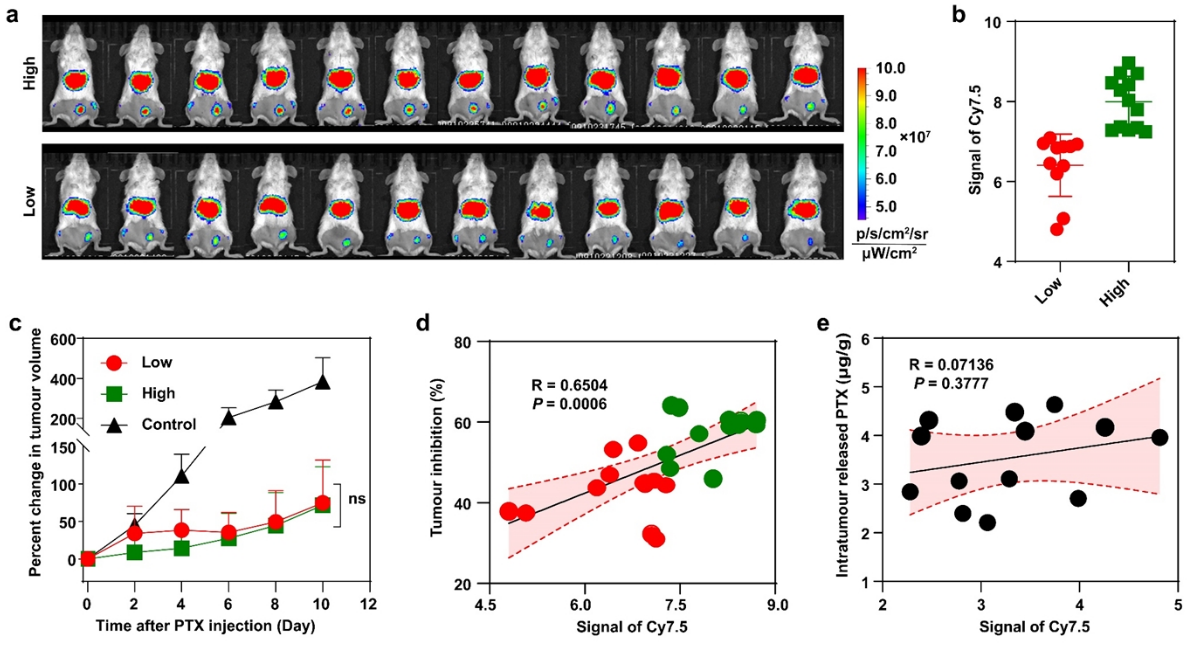

Supplementary Fig. 26. Accumulation fails to predict efficacy of therapeutic nanoparticle PDPA-PTX. (a) Fluorescence images of 4T1 orthotopic tumour-bearing mice were captured at 24 h post-injection of BiRN (20 mg kg^{-1}). Cy7.5: $\lambda_{ex}/\lambda_{em}$ = 780/840 nm (*n* = 12 biologically independent mice). **(b)** Cy7.5 fluorescence signal of 4T1 orthotopic tumour-bearing mice in **a.** Data are presented as mean ± s.d. (*n* = 12 biologically independent mice). **(c)** Tumour progression in 4T1 orthotopic tumours ranked according to low and high accumulation. Data are shown as mean ± s.d. (PBS group, *n* = 8 biologically independent mice; other groups, *n* = 12 biologically independent mice). n.s., not significant, one-way analysis of variance (ANOVA). **(d)** Correlation between Cy7.5 signal and 4T1 tumour progression presented as tumour inhibition percentage (*n* = 24 biologically independent mice). R and *P* values were derived using a simple linear regression model. The error band in red shows the 95% confidence intervals of the fitted line by two-sided Student t-test analysis.. (e) Correlation between intracellular nanoparticle accumulation and intratumoural levels of total PTX at 48 h post-injection of BiRN and PDPA-Cy5 (PTX, 10 mg kg^{-1}). R and *P* values were derived using a simple linear regression model. The error band in red shows the 95% confidence intervals of the fitted line by two-sided Student t-test analysis..

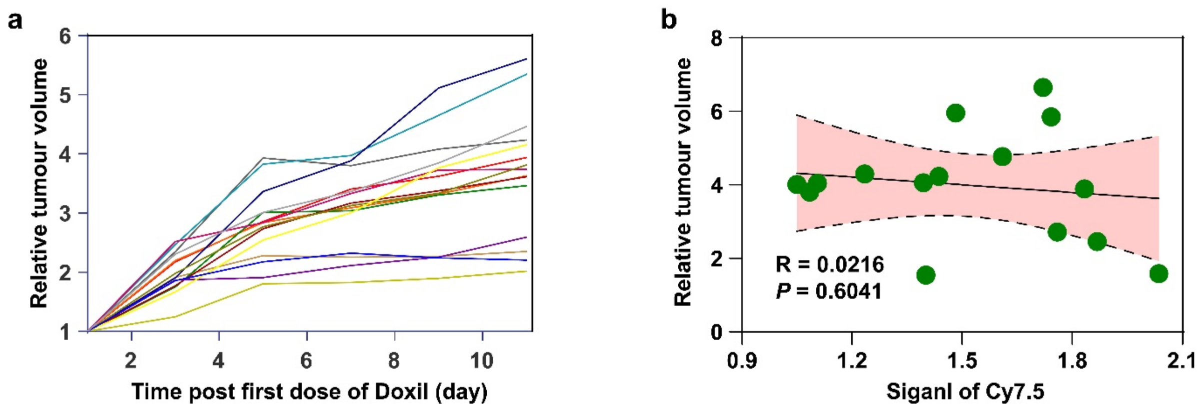

Supplementary Fig. 27. Prediction of 4T1 tumour response to doxorubicin liposome using BiRN technology. (a) Individual tumour growth curves of 4T1 tumour-bearing mice after treatment with doxorubicin liposome (n = 15 biologically independent mice). (**b**) Correlation between Cy7.5 signals and relative tumour volumes (R = 0.1414). R and P values were derived using a simple linear regression model. The error band in red shows the 95% confidence intervals of the fitted line by two-sided Student t-test analysis.

2. Supplementary Tables

Supplementary Table 1. Characterization of PEG_{114}-*b*-$PDPA_{80}$-Dye_{n} copolymers and the resulting nanoprobes.

**Copolymers**	**Feed ratio (Dye: AMA)**	**Conjugation efficiency (%)[a]**	**R_F (F_{max}/F_{min})**	**pH_t[b]**	**$\Delta pH_{10\text{-}90\%}$**	**Functions**
PDPA-BDP_2	2.25 : 3	90.8	104	6.25	0.20	OFF-ON module of $BiRN_{Vis}$[c]
PDPA-$Cy5_3$	4.5 : 3	78.1	100	6.27	0.23	OFF-ON module of $BiRN_{NIR}$[d]
PDPA-$Cy3.5_1$	1.5 : 3	90.4	1.37	6.31	0.24	always-ON module of $BiRN_{Vis}$
PDPA-$BDP650_1$	1.5 : 3	72.5	9.24	6.29	0.22	always-ON module of $BiRN_{Vis}$
PDPA-$Cy7.5_1$	1.5 : 3	70.3	7.78	6.26	0.20	always-ON module of $BiRN_{NIR}$
PDPA-$ICG_{0.5}$	0.75 :3	69.9	9.92	6.28	0.24	always-ON module of $BiRN_{NIR}$

[a] Conjugation efficiency were determined by UV spectrophotometer; [b] pH_t were determined at 37 °C.

[c] $BiRN_{Vis}$ was encoded with fluorophores in the visible window.

[d] $BiRN_{NIR}$ was encoded with fluorophores in the near-infrared window.

Supplementary Table 2. Characterization of different BiRN nanoprobes.

BiRNs	Dye pairs	Molar ratio (%)	R_F (F_{max}/F_{min})	pH_t	$\Delta pH_{10-90\%}$	Applications
$BiRN_{NIR}$	Cy5	90	111	6.28	0.21	in vivo imaging
	Cy7.5	10	1.03	–	–	
	Cy5	95	103	6.28	0.25	in vivo imaging
	ICG	5	1.12	–	–	
$BiRN_{Vis}$	BDP FL	60	121	6.27	0.24	cell imaging<br>flow cytometry<br>intravital imaging
	Cy3.5	40	1.07	–	–	
	BDP FL	90	116	6.27	0.21	cell imaging<br>flow cytometry<br>intravital imaging
	BDP 650	10	1.09	–	–	
