# Supplementary material for: Quantitative imaging of intracellular nanoparticle exposure enables prediction of nanotherapeutic efficacy
Source: Nat Commun. 2021 Apr 22;12:2385. doi: 10.1038/s41467-021-22678-z (PMC8062465; doi:10.1038/s41467-021-22678-z)
Supplement: Supplementary file 2 — Description of Additional Supplementary Files [file 41467_2021_22678_MOESM2_ESM.docx]

**Description of Additional Supplementary Data**

**File name:** Supplementary movie 1

Description: Real-time internalization and activation of BiRN in A549 tumour cells. Images acquisition was performed as soon as the addition of the culture medium containing BiRN_Vis_ (100 μg mL^-1^) into cell culture dish, followed by real-time imaging for 30 min at 1 frame per minute.

**File name:** Supplementary movie 2

Description: Pulse-chase imaging was exploited to investigate the real-time intracellular transport of nanoparticles in Panc02 pancreatic cancer cells. After pulsed with BiRN_Vis_ (100 μg mL^-1^), Panc02 cells were chased by confocal imaging at 37 ºC for 30 min. Images acquisition was performed for 30 min at 1 frame per 3 minute.

**File name:** Supplementary movie 3

Description: 3D imaging of microdistribution of BiRN in 4T1 tumours in living animals. Imaging studies were carried out in 4T1 xenograft tumours grown in dorsal window chambers, mice were anesthetized on a heated microscope stage and 100 μL of the BiRN_Vis_ (40 mg kg^-1^) were administered intravenously, followed by imaging (1 μm per stack, 100 stacks) at 3 h post-injection.
